# Intrinsically disordered substrates dictate SPOP subnuclear localization and ubiquitination activity

**DOI:** 10.1016/j.jbc.2021.100693

**Published:** 2021-04-22

**Authors:** Emery T. Usher, Nafiseh Sabri, Roman Rohac, Amie K. Boal, Tanja Mittag, Scott A. Showalter

**Affiliations:** 1 Department of Biochemistry and Molecular Biology, Pennsylvania State University, University Park, Pennsylvania, USA; 2 Department of Structural Biology, St Jude Children’s Research Hospital, Memphis, Tennessee, USA; 3 Department of Chemistry, Pennsylvania State University, University Park, Pennsylvania, USA

**Keywords:** diabetes, multivalency, Pdx1, SPOP, ubiquitination, structure, AR, androgen receptor, DAPI, 4′,6-diamidino-2-phenylindole, DAXX, death domain-associated protein 6, FA, fluorescence anisotropy, IDRs, intrinsically disordered regions, LLPS, liquid–liquid phase separation, MATH, meprin and tumor necrosis factor receptor-associated factor homology, Ni-NTA, nickel-nitrilotriacetic acid, PBST, Triton X-100 in PBS, Pdx1, pancreatic and duodenal homeobox 1, Puc, puckered protein, SB, SPOP-binding, SBM1, SB motif 1, SBM2, SB motif 2, SPOP, speckle-type POZ protein

## Abstract

Speckle-type POZ protein (SPOP) is a ubiquitin ligase adaptor that binds substrate proteins and facilitates their proteasomal degradation. Most SPOP substrates present multiple SPOP-binding (SB) motifs and undergo liquid–liquid phase separation with SPOP. Pancreatic and duodenal homeobox 1 (Pdx1), an insulin transcription factor, is downregulated by interaction with SPOP. Unlike other substrates, only one SB motif has previously been reported within the Pdx1 C-terminal intrinsically disordered region (Pdx1-C). Given this difference, we aimed to determine the specific mode of interaction of Pdx1 with SPOP and how it is similar or different to that of other SPOP substrates. Here, we identify a second SB motif in Pdx1-C, but still find that the resulting moderate valency is insufficient to support phase separation with SPOP in cells. Although Pdx1 does not phase separate with SPOP, Pdx1 and SPOP interaction prompts SPOP relocalization from nuclear speckles to the diffuse nucleoplasm. Accordingly, we find that SPOP-mediated ubiquitination activity of Pdx1 occurs in the nucleoplasm and that highly efficient Pdx1 turnover requires both SB motifs. Our results suggest that the subnuclear localization of SPOP–substrate interactions and substrate ubiquitination may be directed by the properties of the substrate itself.

Regulation of protein stability is a critical determinant of cellular health and function. In pancreatic β cells, the transcription factor pancreatic and duodenal homeobox 1 (Pdx1; also known as glucose-sensitive factor (1), insulin promoter factor 1 (2), insulin upstream factor 1 (3), and islet/duodenum homeobox 1 (4)) modulates insulin production in response to blood-glucose levels (5, 6). In addition to its role in maintaining glucose homeostasis, Pdx1 is also critical to pancreatic development (7, 8, 9) and β cell differentiation (10). Given the critical roles of Pdx1 in overall pancreatic health, Pdx1 mutation and dysregulation is predictably associated with diabetic phenotypes (7, 11). Among the regulatory signals that control Pdx1 stability and function is a degradation pathway wherein Pdx1 associates with a ubiquitin ligase adaptor, speckle-type POZ protein (SPOP).

SPOP recognizes the disordered C terminus of Pdx1 in conditions of low glucose in the β cell (12, 13). Unlike Pdx1, which functions almost exclusively in β cells, SPOP is found across many tissue types and interacts with numerous substrates (14). SPOP recruits the Cullin3-RING ubiquitin ligase, which facilitates the polyubiquination of SPOP-bound substrates (15). Upon binding to the SPOP–Cullin3-RING ligase complex, Pdx1 is ubiquitinated and degraded (12) and thus cannot activate transcription. Of note, mutations in SPOP that affect substrate binding are associated with prostate and endometrial cancers, among others (16, 17, 18, 19), and so the study of SPOP and its interacting partners has broad implications across many fields.

In addition to a DNA-binding domain, Pdx1 contains two intrinsically disordered regions (IDRs) that mediate many protein–protein interactions (Fig. 1*A*). Notably, many documented diabetes-linked mutations exist within the Pdx1 IDRs, not the DNA-binding domain (Table S1) (11, 20). SPOP is composed of three domains: the meprin and tumor necrosis factor receptor-associated factor homology (MATH) substrate-interaction domain and two dimerization domains, bric à brac, tramtrack, broad complex and bric à brac, tramtrack, broad complex and C-terminal Kelch (Fig. 1*B*). SPOP oligomerizes through sequential dimerization events (21), which serves to enhance apparent affinity for substrates by the display of multiple MATH domains (Fig. 1*B*) (22). To this end, SPOP substrates tend to present multiple binding motifs that individually have relatively weak affinities for SPOP, yet several motifs in a single substrate contribute to a substantially strengthened binding interaction to oligomeric SPOP (Fig. 1*C*) (14). This phenomenon has been demonstrated for SPOP substrates transcriptional activator GLI3, androgen receptor (AR), and death domain-associated protein 6 (DAXX) (21, 22, 23).

**Figure 1:**
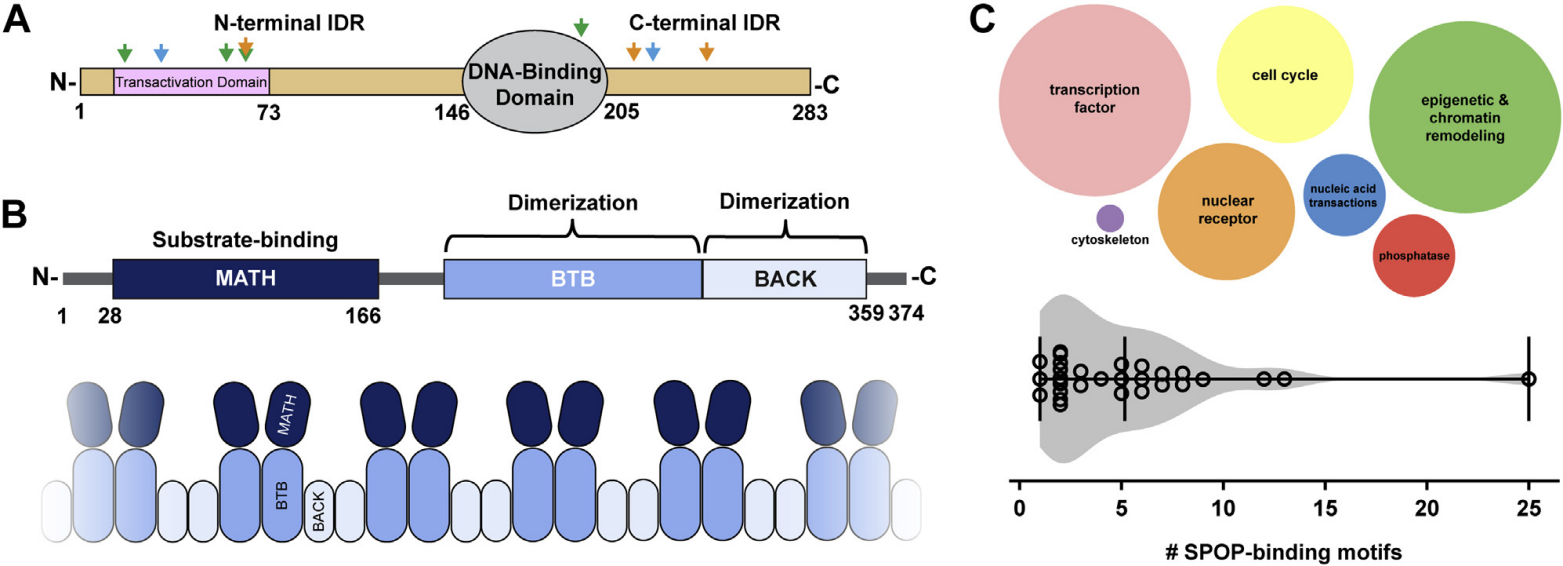
**Pdx1 and SPOP contain distinct domains that contribute to their function.***A*, domain architecture of Pdx1. The N-terminal intrinsically disordered region (IDR) contains a transactivation domain required for Pdx1 transcription factor activity. The DNA-binding domain is a canonical homeodomain and binds A-box DNA motifs. The C-terminal IDR associates with SPOP. The *arrows* represent the locations of Pdx1-assiociated mutations found in patients with type 2 diabetes (*green*), Mature-Onset Diabetes of the Young 4 (*blue*), and Mature-Onset Diabetes of the Young 3 (*orange*). *B*, domain architecture of SPOP (*upper panel*). The MATH domain binds SPOP-binding motifs in substrates, and the bric à brac, tramtrack, broad complex and bric à brac, tramtrack, broad complex and C-terminal Kelch domains facilitate sequential homodimerization to promote concentration-dependent higher-order assembly (*lower panel*). *C*, SPOP contains various substrates, in addition to Pdx1, that are integral to many regulatory processes. Each *circle* represents the relative number of known SPOP substrates in each cellular role (*top panel*). SPOP substrates contain varied numbers of predicted SPOP-binding motifs (14). MATH, meprin and tumor necrosis factor receptor-associated factor homology; Pdx1, pancreatic and duodenal homeobox 1; SPOP, speckle-type POZ protein.

Given the numerous and diverse substrates under regulatory control by SPOP, including Pdx1, we must determine whether SPOP recognizes all substrates by the same mechanism or whether substrate-encoded differences result in their prioritization by SPOP. SPOP has recently been reported to recruit substrates *via* phase separation, which requires multivalent interaction (21, 23, 24). Given that only one SPOP-binding (SB) motif has been identified in Pdx1, we tested whether Pdx1 and SPOP engage and function in phase-separated compartments in cells and found that the two do not phase separate together. Instead, Pdx1 relocalizes SPOP to the nucleoplasm. To test whether this recruitment is indeed the result of the substrate valence for SPOP, we characterized the interaction of Pdx1-C with SPOP *in vitro* and found evidence for a second SB motif in Pdx1 that contributes to high-affinity binding but no additional motifs that would mediate the formation of three-dimensional networks of complexes.

Notably, neither SB motif in Pdx1 conforms to the established SB consensus sequence (25), but high-resolution structural characterization suggests that the Pdx1 SB motifs share a binding mode with other substrates. Finally, we find that the second SB motif is not required to draw SPOP out of nuclear speckles but that it does contribute to Pdx1 ubiquitination levels. Together, our results provide insight into Pdx1 turnover *via* SPOP and present the first example of a non–phase-separating SPOP substrate and the implications of such biophysical behavior for substrate prioritization.

## Results

### Interaction of SPOP and Pdx1 in cells promotes SPOP relocalization

SPOP derives its name from the observation of SPOP localization to membraneless organelles within the nucleus. Specifically, SPOP colocalizes with nuclear speckles but has also been reported in other subnuclear compartments (24, 26). The nuclear speckles that house SPOP in the absence of high concentrations of substrate are liquid-like in nature (21). The SPOP substrate DAXX typically localizes to promyelocytic leukemia bodies (27). When coexpressed in cells, however, SPOP and DAXX relocalize to liquid-like bodies that are distinct from nuclear speckles and promyelocytic bodies. Said new SPOP-DAXX bodies serve as the sites of ubiquitination activity (23).

Consistent with the observation of SPOP-mediated substrate ubiquitination within liquid-like bodies, liquid–liquid phase separation (LLPS) is gaining traction as a mechanism underlying compartmentalization of biological activities, dysregulation of which can result in disease pathologies (28, 29). Multivalent protein–protein interactions mediate LLPS, and increasing valence within each protein enhances the driving force for phase separation (30). Higher-order oligomerization of SPOP and the associated multivalency for its substrates, which themselves present several SB motifs, also drive SPOP–substrate phase separation.

**Figure 2:**
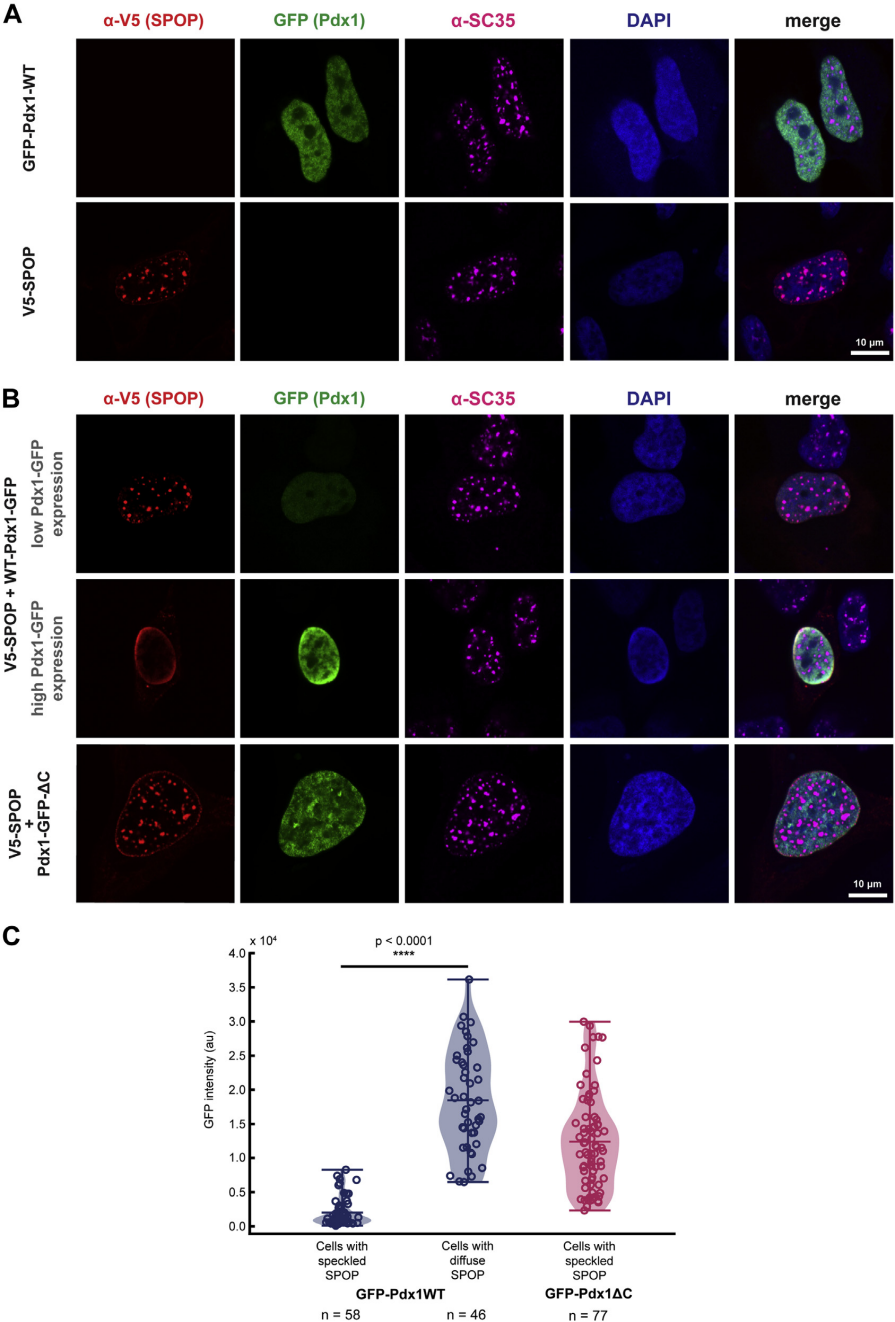
**SPOP and Pdx1 do not phase separate in cells.***A*, when transfected alone, GFP–Pdx1 (*green*) localizes to the nucleus (marked by DAPI, *blue*) and does not perturb the formation of or partition to nuclear speckles (*magenta*) (*upper panel*). V5-SPOP (*red*) localizes to nuclear speckles, which is consistent with previous reports (*lower panel*). *B*, V5-SPOP localizes to nuclear speckles in cells expressing low levels of WT–Pdx1 (*top panel*). Cells that express high levels of WT–Pdx1 have primarily diffuse V5-SPOP (*middle panel*). Deletion of the C-terminal IDR of Pdx1 (Pdx1ΔC) decouples SPOP localization from Pdx1 expression levels (*bottom panel*). *C*, quantification of GFP intensity as a function of SPOP localization for each Pdx1–GFP variant. Data points represent individual cells from at least three biological replicates. Significance was determined using a two-tailed Student’s *t* test. IDR, intrinsically disordered region; Pdx1, pancreatic and duodenal homeobox 1; SPOP, speckle-type POZ protein.

Given that only one SB motif was known in Pdx1, we set out to assess whether Pdx1 and SPOP undergo phase separation in cells. We overexpressed GFP–Pdx1 and V5-tagged SPOP in HeLa cells, performed immunostaining, and observed the cellular localization of the proteins by confocal fluorescence microscopy. In contrast to other SPOP substrates (23), we find that GFP–Pdx1 does not localize to punctate structures in cells (Fig. 2*A*, top panel). As expected, V5-SPOP localizes to nuclear speckles, which are marked by staining for a nuclear speckle scaffold protein (serine/arginine repetitive matrix protein 2) using the SC-35 antibody (31) (Fig. 2*A*, lower panel). However, when GFP–Pdx1–WT and V5-SPOP were coexpressed, SPOP relocalized from nuclear speckles into the diffuse nucleoplasm where GFP–Pdx1 resides. Importantly, Pdx1 did not undergo observable partitioning to any nuclear body (Fig. 2*B*).

Furthermore, SPOP relocalization appears to depend on the concentration of GFP–Pdx1. In cells expressing GFP–Pdx1 at high levels (as assessed by high GFP intensity), SPOP is diffuse, whereas in cells that express GFP–Pdx1 at low levels, SPOP localizes to nuclear speckles (Fig. 2*B*). This result suggests that there exists a substrate concentration threshold above which SPOP is bound and relocalized by the substrate protein. To ensure that SPOP relocalization is dependent on interaction with Pdx1, we repeated the protein localization experiments using a construct of GFP–Pdx1 lacking its C terminus (GFP–Pdx1ΔC). Indeed, we found that a Pdx1 construct that is incapable of interacting with SPOP is also incapable of drawing SPOP out of nuclear speckles (Fig. 2*B*). To our knowledge, this is the first demonstration of SPOP redistribution from nuclear speckles toward interaction with a substrate diffusely in the nucleus instead of in a nuclear body.

### Two motifs in the Pdx1 C terminus interact with SPOP

Our results suggested that the valence of one Pdx1 for SPOP was not sufficient to mediate phase separation. However, given that SB motifs can have weak affinities and some sequence variability, we set out to identify potential additional SB motifs in Pdx1 that might point to a different mechanism of SPOP redistribution. Given a single motif in the Pdx1 C terminus (32, 33), we probed this interaction by NMR spectroscopy.

**Figure 3:**
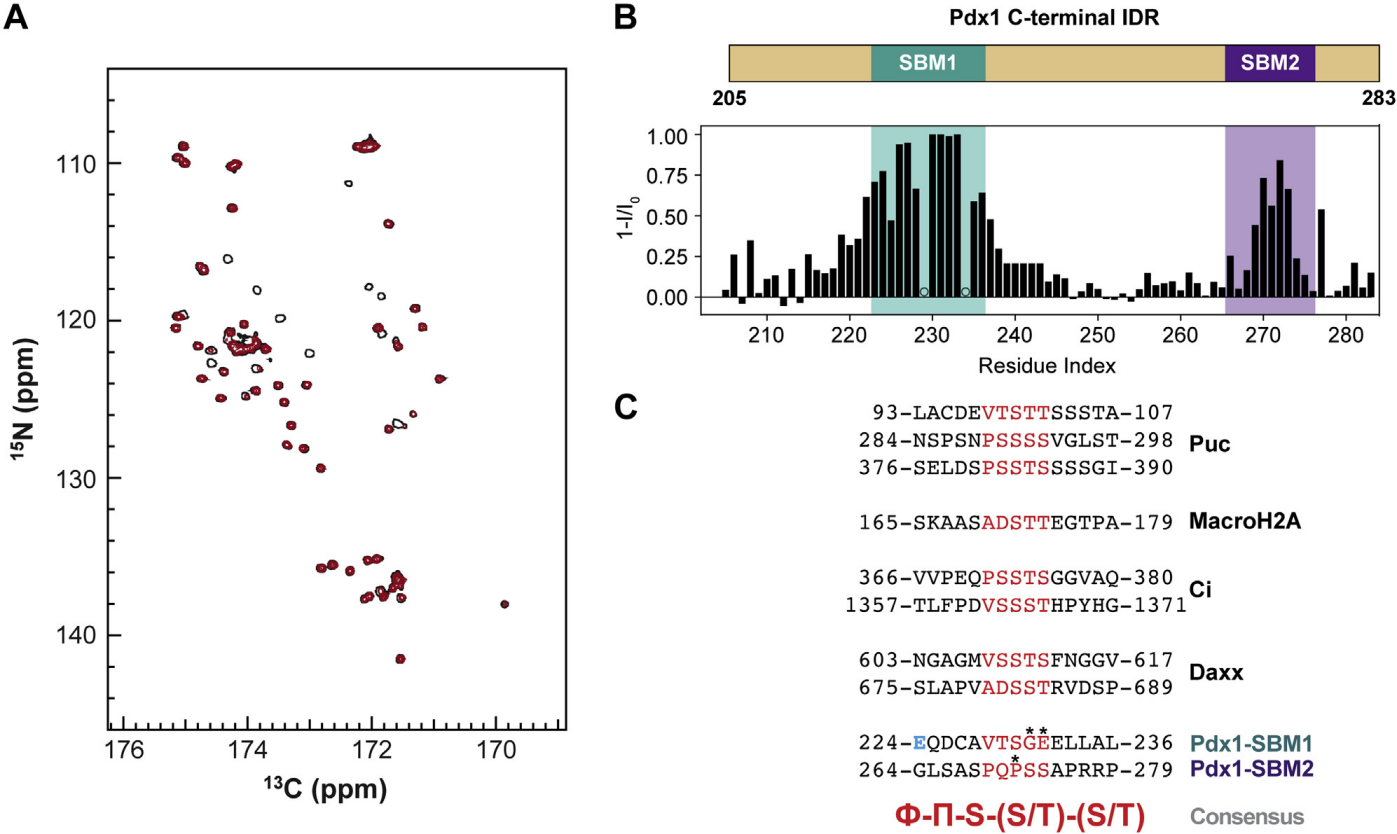
**SPOP–MATH interacts with Pdx1 by two motifs in the C terminus.***A*, addition of 4 M equivalents of SPOP–MATH into ^13^C, ^15^N–Pdx1-C (residues 204–283) monitored by the ^13^C, ^15^N–(HACA)CON NMR experiment collected at 500 MHz and 25 °C. *Hollow black* resonances represented unbound ^13^C, ^15^N–Pdx1-C and *filled red* resonances show SPOP-bound ^13^C, ^15^N–Pdx1-C. *B*, intensity changes of ^13^C, ^15^N–Pdx1-C resonances after the addition of SPOP–MATH plotted as a function of Pdx1 residue number. SBM1 and SBM2 are indicated by *bars* above the sequence in *teal* and *purple*, respectively. Residues marked with *circles* represent missing data points due to peak overlap. *C*, alignment of a subset of characterized SPOP substrates. The SPOP-binding sequences are colored in *red*, and the consensus sequence is shown in *red below*; a small, hydrophobic residue (Ф) and a polar residue (П). Consensus sequence mismatches, which have only been found in Pdx1, are marked above by *asterisks*. MATH, meprin and tumor necrosis factor receptor-associated factor homology; Pdx1, pancreatic and duodenal homeobox 1; SBM1, SB motif 1; SBM2, SB motif 2; SPOP, speckle-type POZ protein.

To identify specific residues on Pdx1 that may interact with SPOP, we used ^13^C direct-detect NMR experiments tailored to the biophysical characterization of IDRs. Such methods allow enhanced resolution over traditional proton-detect methods for cases, such as Pdx1-C, wherein spectral crowding and degeneracy in unique chemical shifts are barriers to data interpretation (34, 35). We collected (HACA)CON spectra of ^13^C, ^15^N-Pdx1-C (residues 204–283) with and without SPOP-MATH (residues 28–166) (Fig. 3*A* and Fig. S1, *A* and *B*). The addition of SPOP led to marked resonance intensity loss in two specific locations: amino acids 220 to 235 (highlighted in teal) and amino acids 265 to 275 (highlighted in purple, Fig. 3*B*). The teal region maps to the fragment of Pdx1 that was already determined by pulldown and crystallography to interact with SPOP, and we interpret the disappearance of resonances here and at the second cluster (purple) as binding to SPOP-MATH. We are confident that the spectral changes are due to direct interaction with SPOP because Pdx1-C shows no evidence of any intramolecular interactions that would otherwise explain the observed resonance intensity changes (36). Importantly, the amino acids in both regions of interest in Pdx1-C are highly conserved across several species, suggesting an evolutionary pressure to retain such motifs (Fig. S1*C*). Thus, we propose that Pdx1-C contains two distinct SB motifs, SB motif 1 (SBM1) and SB motif 2 (SBM2), the sequences of which are shown in alignment with other SPOP substrates in Figure 3*C*. We did not see evidence for any additional SB motifs.

**Figure 4:**
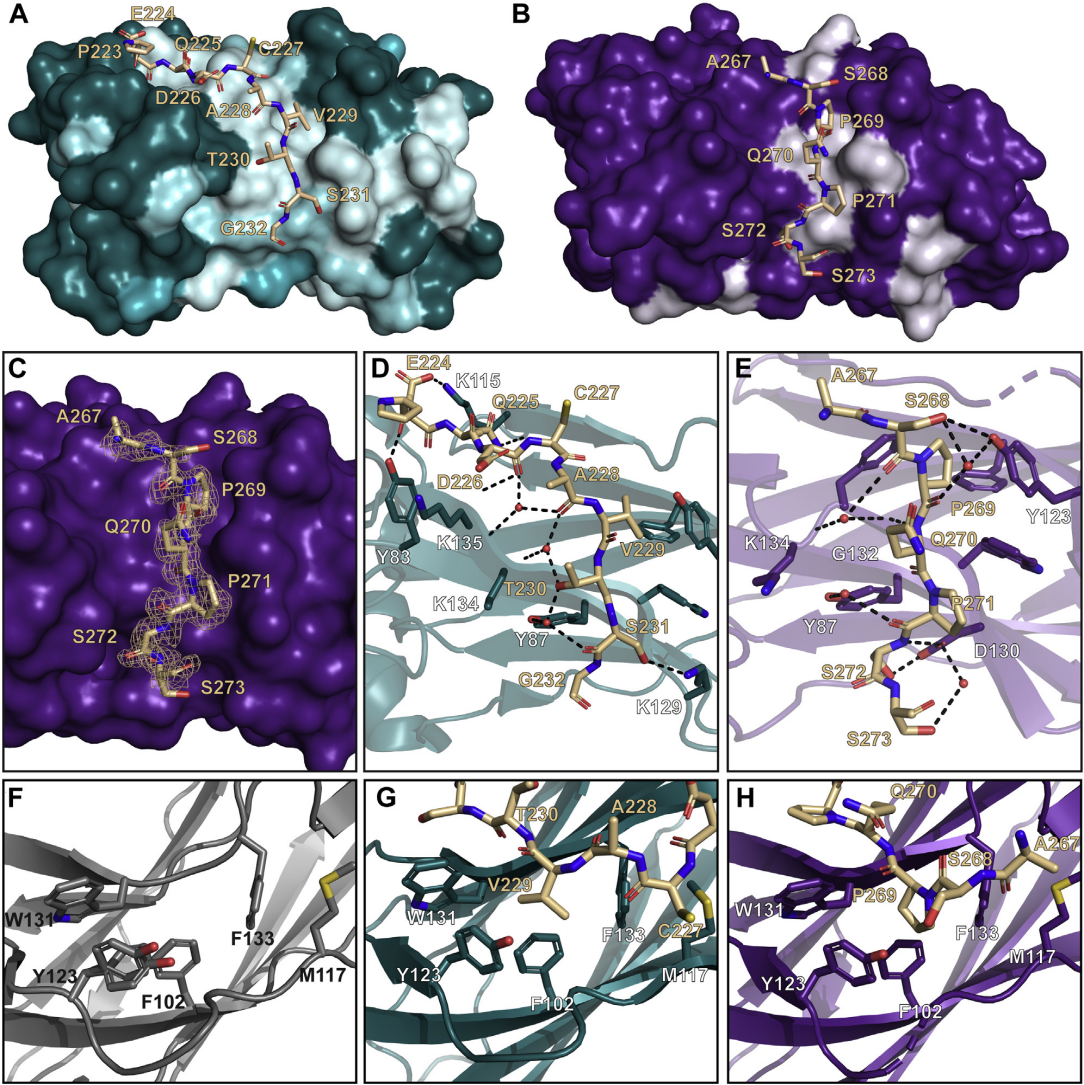
**Both binding sites within Pdx1 interact with the same groove on SPOP.***A*, per-residue intensity changes of ^15^N–SPOP–MATH resonances after the addition of 4-fold molar excess Pdx1–SBM1 peptide plotted on the surface of the Pdx1–SPOP–MATH cocrystal structure from Ostertag *et al.*, 2018 (PDB ID: 6F8F). *Dark teal* to *white* indicates lowest to highest intensity change. *B*, per-residue intensity changes of ^15^N–SPOP–MATH resonances after the addition of 4-fold molar excess Pdx1–SBM2 peptide are plotted on the surface of SPOP. *Dark purple* to *white* indicates lowest to highest resonance intensity change. *C*, surface representation of SPOP–MATH (*purple*) bound to peptide of Pdx1–SBM2 (*tan*) with polder map contoured at + 3σ (55). *D*, hydrogen-bonding scheme of Pdx1–SBM1 (*tan*) and SPOP–MATH (*teal*) (PDB ID: 6F8F). Heteroatoms are depicted in *blue* (nitrogen), *red* (oxygen), and *yellow* (sulfur). Pdx1–SBM1 forms several direct and water-mediated hydrogen bonds to SPOP–MATH. Backbone and side-chain H-bonds (*black dashed lines*) stabilize Pdx1–SBM1 in the binding cleft of SPOP. Pdx1 residues are labeled in *tan*, and SPOP residues are labeled in *white*. *E*, hydrogen-bonging scheme of Pdx1–SBM2 (*tan*) and SPOP–MATH (*purple*). Pdx1–SBM2 forms direct and water-mediated hydrogen bonds to SPOP, similar to those found in existing structures of SPOP–MATH–substrate complexes. Pdx1 residues are labeled in *tan*, and SPOP residues are labeled in *white*. *F*, substrate-free (apo) SPOP–MATH (*gray ribbon*). Heteroatoms are *blue* (nitrogen), *red* (oxygen), and *yellow* (sulfur). Five large, hydrophobic residues including F102, M117, Y123, W131, and F133 constitute a hydrophobic binding pocket. *G*, cocrystal structure of Pdx1–SBM1 (*tan sticks*) bound to SPOP–MATH (*teal ribbon*). V229 (position 1 of the consensus SPOP-binding motif) of Pdx1 sits within the hydrophobic pocket. *H*, cocrystal structure of Pdx1–SBM2 (*tan sticks*) bound to SPOP–MATH (*purple ribbon*). P269 sits in the hydrophobic binding pocket and anchors the peptide despite mismatches from the consensus SPOP-binding motif. MATH, meprin and tumor necrosis factor receptor-associated factor homology; Pdx1, pancreatic and duodenal homeobox 1; SBM1, SB motif 1; SBM2, SB motif 2; SPOP, speckle-type POZ protein.

Addition of individual SBM1 (Pdx1 residues 224–236) and SBM2 (Pdx1 residues 265–283) peptides into ^15^N-SPOP-MATH showed that the resulting spectral changes are primarily localized to the substrate-binding groove (Fig. 4, *A* and *B* and Fig. S1, *D* and *E*), excluding the possibility that one SB motif preferentially interacts with another face of SPOP-MATH. Similarly, when SBM1 and SBM2 peptides were added to ^15^N-SPOP-MATH in equimolar ratios, the spectral changes were localized to the substrate-binding groove and largely resembled those observed with the SBM1 peptide alone (Fig. S1*F*). This suggests a higher SB affinity of Pdx1–SBM1 than Pdx1–SBM2 (as mentioned previously). The spectral changes correspond very well with the location of the Pdx1–SBM1 peptide in the published cocrystal structure (Fig. 4*A*) (33). Furthermore, we posit that the intensity changes on the SPOP surface that extend beyond the binding groove may be explained by transient contacts with SPOP that could confer additional stability and/or affinity.

### The novel SB motif in Pdx1 resembles known motifs despite consensus sequence deviation

Upon identification of the novel binding site for SPOP within Pdx1-C, we sought to understand the molecular basis for its interaction with SPOP. Owing to unfavorable intermediate exchange kinetics and the resulting line broadening, solution NMR was not a viable strategy for structure determination. We therefore pursued crystallography of the complex between the Pdx1 peptide (human protein residues 265–283) and SPOP-MATH (Fig. 4*C*). We also present, to our knowledge, the first X-ray structure of SPOP-MATH without bound substrate. X-ray data collection and refinement statistics may be found in Table S2.

The original consensus SB motif (Fig. 3*C*) was established based on the substrate sequence conservation and structural features of a substrate-binding groove that spans one of the central β-sheets in SPOP-MATH. One side is largely nonpolar with a small cavity to accommodate the aliphatic (Φ) consensus residue. On the other side, the groove is lined with polar side chain and backbone functional groups. Many of the latter arise from unsatisfied H-bonding groups in the top strand in the central β-sheet in SPOP-MATH. In the structures of puckered protein (Puc), core histone Macro-H2A, cubitus interruptus, or DAXX peptides in complex with SPOP, the peptide side chains in sites 3 to 5 of the consensus motif (conserved as Ser or Thr in non-Pdx1 substrates characterized previously) are highly complementary to the polar end of the groove (25). Most of these side chains H-bond—either directly or *via* water-mediated contacts—to SPOP. However, many other sequence-independent substrate backbone contacts are also present, and these may confer the ability to bind nonstandard sequences, such as those found in Pdx1 SBM1 and SBM2.

The recent X-ray structure of Pdx1–SBM1 bound to SPOP-MATH provided the first atomic-level characterization of an SPOP substrate with two deviations from the consensus SB motif (33) (Fig. 3*C*). Despite these differences, the SBM1 peptide lies in the deep substrate-binding groove of SPOP, and this binding mode is stabilized by a network of direct and water-mediated hydrogen bonds. Pdx1–SBM1 forms ten H-bonds to SPOP: six *via* its backbone and four *via* its side chains (Fig. 4*D*). Notably, only two of the side chain–mediated hydrogen bonds involve residues within the consensus binding motif, T230 and S231, positions 2 and 3, respectively. The other two intermolecular side chain H-bonds lie upstream of the consensus SB motif (Table S3).

The 1.7 Å resolution cocrystal structure of Pdx1–SBM2 bound to SPOP–MATH (Fig. 4*B*) reported here places the Pdx1 peptide in the canonical substrate-binding site and is consistent with NMR titration data (Fig. S1*F*) (25, 33, 37, 38). Pdx1–SBM2 also deviates from the consensus SB motif sequence (Fig. 3*C*), containing a Pro at site 3 instead of a Ser. Pdx1–SBM2 also contains a Pro at position 1. These side chains at the beginning and middle of the consensus binding motif are clearly identifiable in the electron density (Fig. S2*A*), making peptide placement unambiguous. As is required for other characterized SPOP substrates, Pdx1–SBM2 harbors a small aliphatic residue in consensus position 1 (P269) (Fig. 4*H*). Similarly, Pdx1–SBM1 V229 (consensus position 1) anchors the peptide in a hydrophobic pocket formed by SPOP residues F102, M117, Y123, W131, and F133 within the binding groove (Fig. 4, *F* and *G*). F102, W131, and F133 are frequently mutated in prostate cancers, which results in reduced affinity of SPOP for substrate proteins (23, 37, 39). Notably, SB motif sequences with a proline as the requisite hydrophobic residue appear to have systematically diminished binding affinities compared with those containing other small aliphatic residues at this position (25).

An analysis of SPOP polar contacts with Pdx1–SBM2 shows several shared features with other substrate complexes, including Pdx1–SBM1 (Fig. S2, *B* and *C*). An approximately equal number of backbone (five) and side chain (four) H-bonds contribute to the stability of the SBM2–SPOP complex. As with Pdx1–SBM1, only two of the side chain contacts involve consensus motif positions, S272 and S273, at positions 4 and 5 (Fig. 4*E*). The other two side chain contacts involve S268, which is N-terminal to the consensus motif and similar to extended interactions between SPOP and Pdx1–SBM1 outside the consensus motif.

Pdx1–SBM2 differs from all other structurally characterized substrate SB motifs as the serine in position 3 of the consensus sequence is not conserved. In Pdx1–SBM1, this serine hydroxyl group hydrogen bonds to K129 on SPOP. In Pdx1–SBM2, a proline resides in position 3, but the peptide is stabilized by H-bonding by the serine residues in positions 4 and 5 (Fig. 4*E*). This could be permitted by unique backbone geometry allowed by the nearby prolines that are absent in other substrates. In the SBM2 complex, the distinctive internal Pro is flanked by four of the five backbone contacts, three of which are unique to SBM2. The Pro in this position could help preorganize the Pdx1 backbone to favor these interactions upon binding.

There is no evidence to suggest that global structural changes contribute substantially to the energetics of binding, as comparison between substrate-bound and substrate-unbound SPOP–MATH structures reveals only minor side chain conformational changes upon substrate binding (Fig. S2, *D* and *E*). In the structure of unbound SPOP–MATH, the electron density maps support modeling of Y123 in two different positions, but in substrate-bound structures, the residue instead adopts a single conformation (Fig. S2*E*). In addition, comparison of the peptide conformations in the Pdx1–SBM1 (Fig. 4*A*) and Pdx1–SBM2 (Fig. 4*B*) structures shows several residues upstream of the consensus motif in the SBM1 structure that are not captured in the SBM2 structure electron density. Specific interactions between Pdx1–E224 and Pdx1–Q225 and SPOP (Fig. S2*F*) may contribute to the enhanced affinity of the SPOP–SBM1 complex.

### Pdx1 binding to SPOP is enhanced by multivalency

SPOP substrates often display multiple SB motifs that contribute to enhanced binding affinity through multivalent contacts (14, 22). SPOP dimerization and subsequent oligomerization *via* its bric à brac, tramtrack, broad complex and bric à brac, tramtrack, broad complex and C-terminal Kelch domains, respectively, serve to increase binding affinity to substrates by presenting multiple MATH domains (21, 40). Multivalency of SPOP–substrate interactions results in avidity effects and longer residence times of substrates on SPOP and promote processivity of ubiquitin conjugation *via* the Cullin-3-RING ligase (22). Accordingly, dimerization- and oligomerization-defective SPOP mutants or deletion of SB motifs impair ubiquitination activity toward substrates DAXX and Puc (23, 25).

With the knowledge that Pdx1 contains two SB motifs, we probed the binding behavior of monomeric SPOP–MATH and oligomeric SPOP_28–359_ toward Pdx1 using competition fluorescence anisotropy (FA) binding experiments. In each titration, we monitored the displacement of a fluorescein-labeled peptide of the SPOP substrate Puc (f-Puc). Puc residues 91 to 106 conform well to the consensus SB motif (Fig. 3*C*) (21) and have a relatively high affinity for SPOP (*K*_D_ = 2.6 μM (Fig. S3*A*)).

**Figure 5:**
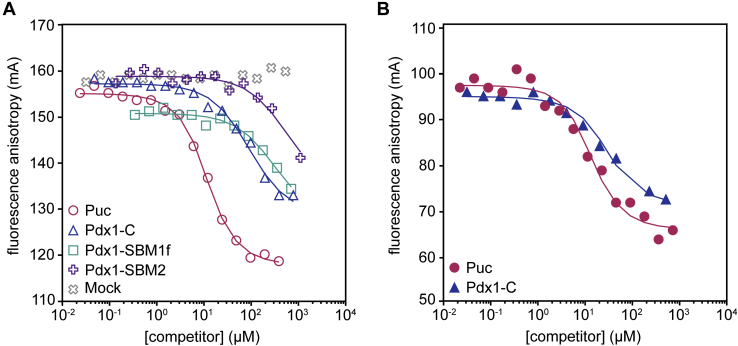
**Multiple binding sites within Pdx1 enhance affinity for SPOP.***A*, competition binding experiments wherein fluorescently labeled Puc peptide (f-Puc) competed for binding to SPOP–MATH in the presence of various competitors: *dark* Puc peptide (*pink circles*, positive control), Pdx1-C (*blue triangles*), Pdx1–SBM1f (*teal squares*), Pdx1–SBM2 (*purple crosses*), or mock peptide (*gray exes*, negative control). *Solid lines* represent complete competitive binding fits (see Experimental procedures), and derived dissociation constants are found in Table S4. *B*, competition binding experiments wherein fluorescently labeled Puc peptide (f-Puc) competed for binding to SPOP_28–359_ with either unlabeled Puc peptide (*pink circles*) or Pdx1-C (*blue triangles*). *Solid lines* are fits to the data for complete competitive binding. Dissociation constants are found in Table S4. MATH, meprin and tumor necrosis factor receptor-associated factor homology; Pdx1, pancreatic and duodenal homeobox 1; SBM1, SB motif 1; SBM2, SB motif 2; SPOP, speckle-type POZ protein.

First, we assessed the binding affinities of each SB motif in Pdx1 to SPOP–MATH using synthetic peptides (Fig. 5*A*). To maximize solubility, the peptide used for FA (Pdx1 residues 224–234) is slightly shorter than the SBM1 peptide used in the NMR titration and is referred to as Pdx1–SBM1f (Table S4). Pdx1–SBM2 binds weakly with a *K*_D_ > 600 μM. Quantitative determination of binding affinity is limited by poor peptide solubility that precludes establishment of a baseline for the fully saturated complex. Pdx1–SBM1f binds with slightly higher affinity, with a *K*_D_ of 140 μM. Given that the lower limit of the *K*_D_ for SBM2 is well in excess of the SBM1 dissociation constant, we assign Pdx1–SBM1 and Pdx1–SBM2 as higher and lower affinity binding sites, respectively.

Interestingly, Pdx1-C, which contains both SB motifs, binds with a higher apparent affinity for SPOP–MATH than the strongest individual SB motif alone. This finding suggests that the enhancement in binding from avidity is strong enough to overcome the inherent competition of these two peptides for interaction with the same SPOP–MATH binding surface. A similar mechanism has been described for polyvalent Sic1 binding to a single site on Cdc4, where the high local concentration of binding motifs around the Cdc4 binding site favors rebinding over Sic1 release (41). Although this is an intriguing kinetic mechanism, enhanced affinity may be thermodynamically driven by favorable chain entropy when both binding motifs exist in the same polypeptide.

We next sought to assess the role of SPOP oligomerization in the SPOP–Pdx1 interaction and saw a near 10-fold enhancement in binding affinity of Pdx1 to oligomeric SPOP_28–359_ compared with SPOP–MATH (Fig. 5*B* and Table S4) in agreement with previous similar observations (22, 23). Concentration-dependent SPOP self-association results in an ensemble of oligomeric species with sizes spanning from dimers to large multimers (21). SPOP substrates DAXX and AR are also multivalent for SPOP; they contain six and seven SB motifs, respectively, which dramatically enhances their affinities for SPOP_28–359_ relative to SPOP–MATH (23). Our finding that Pdx1–SPOP multivalency drives a high-affinity binding interaction suggests that, despite having fewer SB motifs, Pdx1 behaves similarly to other SPOP substrates.

### SPOP-linked ubiquitination of Pdx1 is linked to their multivalent interaction

**Figure 6:**
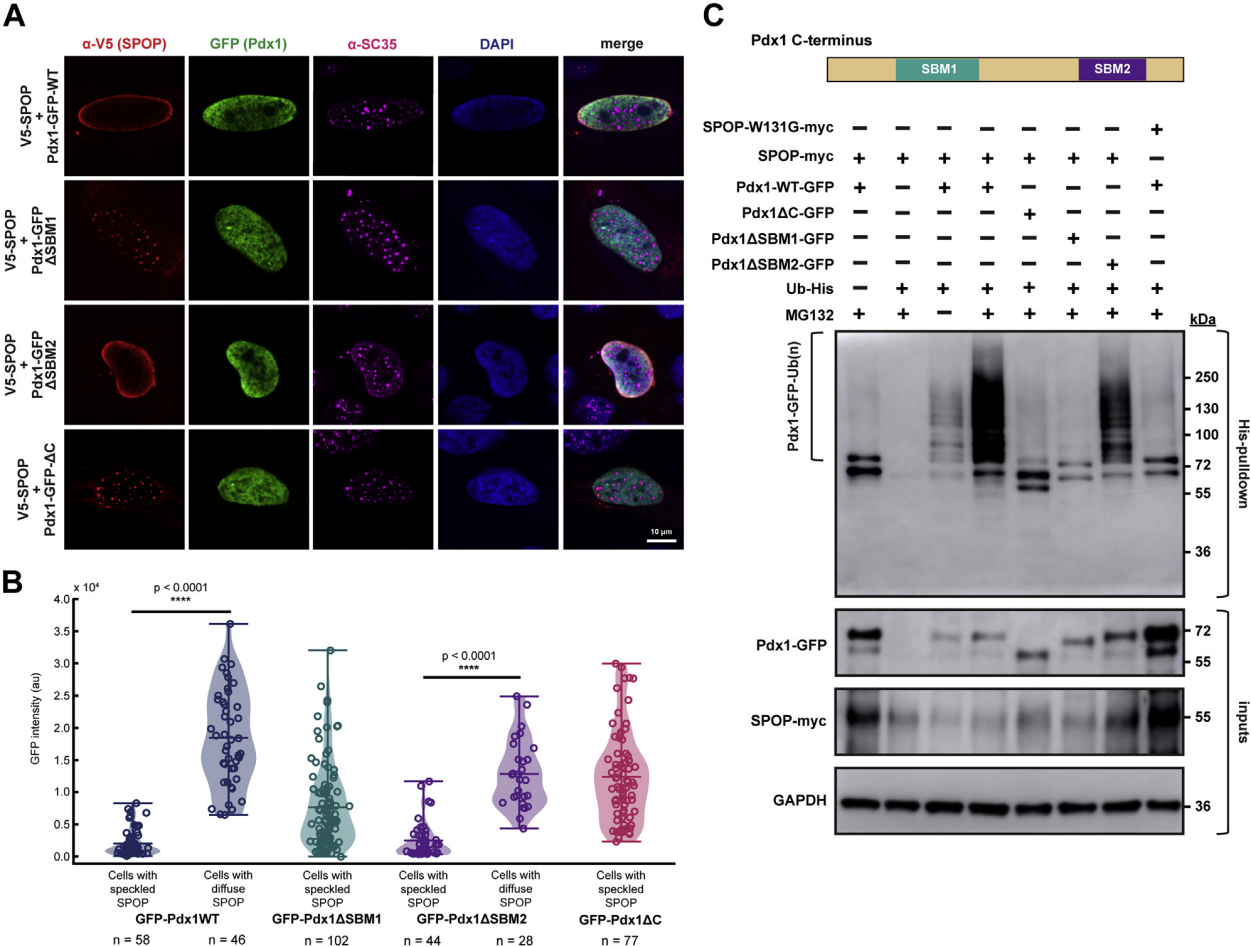
**SPOP relocalization and ubiquitination are concomitant with substrate interaction.***A*, GFP–Pdx1 (*green*) variants relocalize SPOP (*red*) from nuclear speckles (*magenta*) only if the Pdx1 motif(s) can substantiate an interaction. HeLa cells were transfected with Pdx1–GFP variant and V5-SPOP and then visualized using immunofluorescence. *B*, quantification of GFP intensity as a function of SPOP localization for each Pdx1–GFP variant. Data points represent individual cells from at least three biological replicates. Significance was determined using a two-tailed Student’s *t* test. Note that the WT and ΔC data in this panel are reproduced from Figure 2*C* for comparison. *C*, GFP–Pdx1–WT is ubiquitinated in cells. Note that the Pdx1 N-terminal IDR contains a stretch of several histidine residues, which permit pulldown of unmodified Pdx1 in addition to the ubiquitinated species. T-REx-293 cells were transfected with plasmids encoding SPOP–Myc and the GFP–Pdx1 variant (Fig. S3*B*). After 24 h, the cells were treated with MG132 or dimethyl sulfoxide for 4 h and then collected for pulldown and immunoblotting. The smeared band migrating above the unmodified Pdx1 band in the pull-down condition is consistent with a heterogeneous population of polyubiquitinated Pdx1. Pdx1 lacking SBM2 shows lower levels of ubiquitination, as shown by lighter discrete bands. Pdx1 lacking SBM1 and the entire C terminus (negative control) were not observably ubiquitinated. The lack of any shift of the Pdx1 band in cells that were expressing the substrate binding-impaired mutant, W131G, implies that the ubiquitination activity is SPOP dependent. IDR, intrinsically disordered region; Pdx1, pancreatic and duodenal homeobox 1; SBM1, SB motif 1; SBM2, SB motif 2; SPOP, speckle-type POZ protein.

From our *in vitro* binding data, which support a cooperative binding model through multivalency, we hypothesized that both SB sites within Pdx1 are required for their interaction in cells. To test this proposal, we cotransfected HeLa cells with plasmids encoding Pdx1 variants (Fig. S3*B*) and V5-SPOP. Deletion of the higher-affinity SBM1 (residues 224–236) decouples SPOP localization from Pdx1 expression. That is, all cells, regardless of GFP–Pdx1–ΔSBM1 levels, contain punctate V5-SPOP (Fig. 6, *A* and *B*). This result suggests that the weaker SB motif (SBM2) alone is insufficient to form a persistent interaction with SPOP in cells. Strikingly, deletion of the weaker motif (SBM2) still enables SPOP relocalization as a function of Pdx1 expression (Fig. 6, *B* and *C*), suggesting that GFP–Pdx1–ΔSBM2 is still competent to bind SPOP to some extent despite the relatively weak affinity of SBM1 alone. As expected, deletion of the full C terminus including both SB motifs (GFP–Pdx1-ΔC) results in the same protein localization as GFP–Pdx1–ΔSBM1, that is, SPOP localizes in nuclear speckles regardless of Pdx1 expression levels (Fig. 6, *A* and *B*).

We next sought to determine whether SPOP-driven ubiquitination of Pdx1 could occur outside of previously implicated droplets. Given the SPOP relocalization phenotype that we observe in cells that express SPOP and Pdx1, we hypothesized that the ubiquitination activity was occurring in the diffuse nucleoplasm and that optimal activity requires both SB sites. To test this hypothesis, we performed in cell ubiquitination assays with GFP–Pdx1 variants. SPOP–myc and GFP–Pdx1 were coexpressed for 24 h in T-REx-293 cells, followed by a 4-h treatment with 20 μM MG132 or dimethyl sulfoxide. Proteins modified with His-ubiquitin were enriched by pulldown on nickel-nitrilotriacetic acid (Ni-NTA) resin.

We observed robust polyubiquitination of Pdx1–WT, which is seen by the smear to a high molecular weight in the pull-down condition (Fig. 6*C*). Pdx1-ΔC, which is incapable of binding to SPOP and does not drive SPOP relocalization, is not ubiquitinated (Fig. 6*C*). Deletion of the stronger SB motif (Pdx1–ΔSBM1), also impedes ubiquitination, as shown by no shift from the unmodified band. Notably, deletion of the weaker SB motif (Pdx1–ΔSBM2) does not completely inhibit ubiquitination but appears to decrease efficiency (Fig. 6*C*). This observation is in agreement with a requirement for SPOP and substrate to localize to the same compartment for efficient ubiquitination to occur. In addition, reduced ubiquitination in Pdx1–ΔSBM2 is consistent with the notion that SPOP–substrate multivalency enhances ubiquitination efficiency. The lack of either of the SB sites in Pdx1 compromises its SPOP-mediated ubiquitination.

In a similar experiment, the cells were not treated with a proteasome inhibitor to observe the SPOP-dependent stability of each Pdx1 variant. As expected, Pdx1–WT is readily degraded, whereas Pdx1–ΔSBM1, Pdx1–ΔSBM2, and Pdx1-ΔC are all more stable relative to the WT protein containing both SB motifs (Fig. S3*D*). Consistent with experiments to monitor the buildup of polyubiquitinated Pdx1, deletion of the weaker SB motif (SBM2) leads to a modest increase in Pdx1 stability but is still subject to greater SPOP-dependent turnover than the deletion of the stronger SB motif (SBM1) (Fig. S3*D*).

## Discussion

Here, we describe a new potential mechanism by which the ubiquitin ligase adaptor SPOP targets and facilitates modification of its substrate proteins. To date, biophysical studies of SPOP–substrate interactions have centered on substrates that contain several SB motifs that support LLPS of SPOP–substrate in cells (22, 23). The pancreatic transcription factor Pdx1, however, contains only two SB motifs. Pdx1 and SPOP do not form phase-separated compartments in cells; the two SB motifs presented by Pdx1 do not efficiently mediate the formation of three-dimensional protein networks that support phase separation. Instead, high levels of Pdx1 draw SPOP out of nuclear speckles and the SPOP-mediated ubiquitination of Pdx1 occurs in the nucleoplasm. Given that SPOP substrates contain highly varied numbers of SB motifs (Fig. 1*C*), we propose that SPOP substrates control their prioritization by determining colocalization with SPOP in nuclear condensates or the nucleoplasm.

### Pdx1 contains two SB motifs that contribute to a high-affinity interaction

Previous studies of Pdx1 and SPOP interaction did not identify a second SB motif in the Pdx1 C terminus. Indeed, there are substantial challenges associated with the site-specific characterization of protein IDRs and their interactions. To overcome this barrier, we used ^13^C direct-detect NMR methods that allow good peak dispersion and spectral resolution compared with traditional proton-detect methods (34, 35). Such methods allowed us to obtain residue-level information about SPOP interaction with the Pdx1 C-terminal IDR and uncover a second, cryptic SB motif. Furthermore, the binding footprint of SPOP on SBM1 may be larger than previously thought. This model may even be expanded to explain certain cancer-linked SPOP mutations that lie outside of the substrate-binding groove and are proposed to increase substrate affinity (14, 16).

Neither Pdx1 SB motif closely adheres to the established SB consensus sequence. The consensus SB motif was initially described based on the experimental characterization of SB motifs in a subset of SPOP substrates (25) that has since grown to include a functionally diverse set of substrates, some of which have motifs that do not conform to the accepted consensus sequence (14). Comprehensive characterization of substrates containing divergent SB motifs, such as Pdx1, is valuable because it is plausible that adherence or departure from this consensus is a determinant of the strength of binding to SPOP. We also demonstrate that both SB motifs in Pdx1 are important for high-affinity binding to SPOP and that each SB motif alone has a dramatically lower affinity (Fig. 5*B* and Table S4). This result is consistent with the findings in other studies that propose that multivalency between the SB motifs in the substrate and the SPOP oligomer is critical for an efficient interaction (22, 23).

### SPOP is relocalized by, but does not phase separate with, low-valence substrate Pdx1

We do not observe *in vitro* phase separation of Pdx1, nor do we observe partitioning of Pdx1 to a new or preformed dense phase in cells. These findings distinguish Pdx1 from other SPOP substrates studied in cells to date (21, 23). Yet, Pdx1 still binds SPOP and exploits multivalency from multiple binding sites to create a high-affinity interaction. In cells, we observe relocalization of SPOP from nuclear speckles toward interaction with Pdx1 in the nucleoplasm, which implies that droplet formation is not required for SPOP-mediated ubiquitination to occur but rather that ubiquitination can happen wherever substrate and SPOP colocalize.

Substrates known to undergo partition into liquid-like compartments alone, such as DAXX or AR, which have six and seven SB sites, respectively, interact with SPOP in phase-separated droplets in cells and undergo robust SPOP-mediated polyubiquitination (23). We observe robust polyubiquitination of Pdx1 (Fig. 6*C*) although it has been previously described to undergo primarily monoubiquitination.

**Figure 7:**
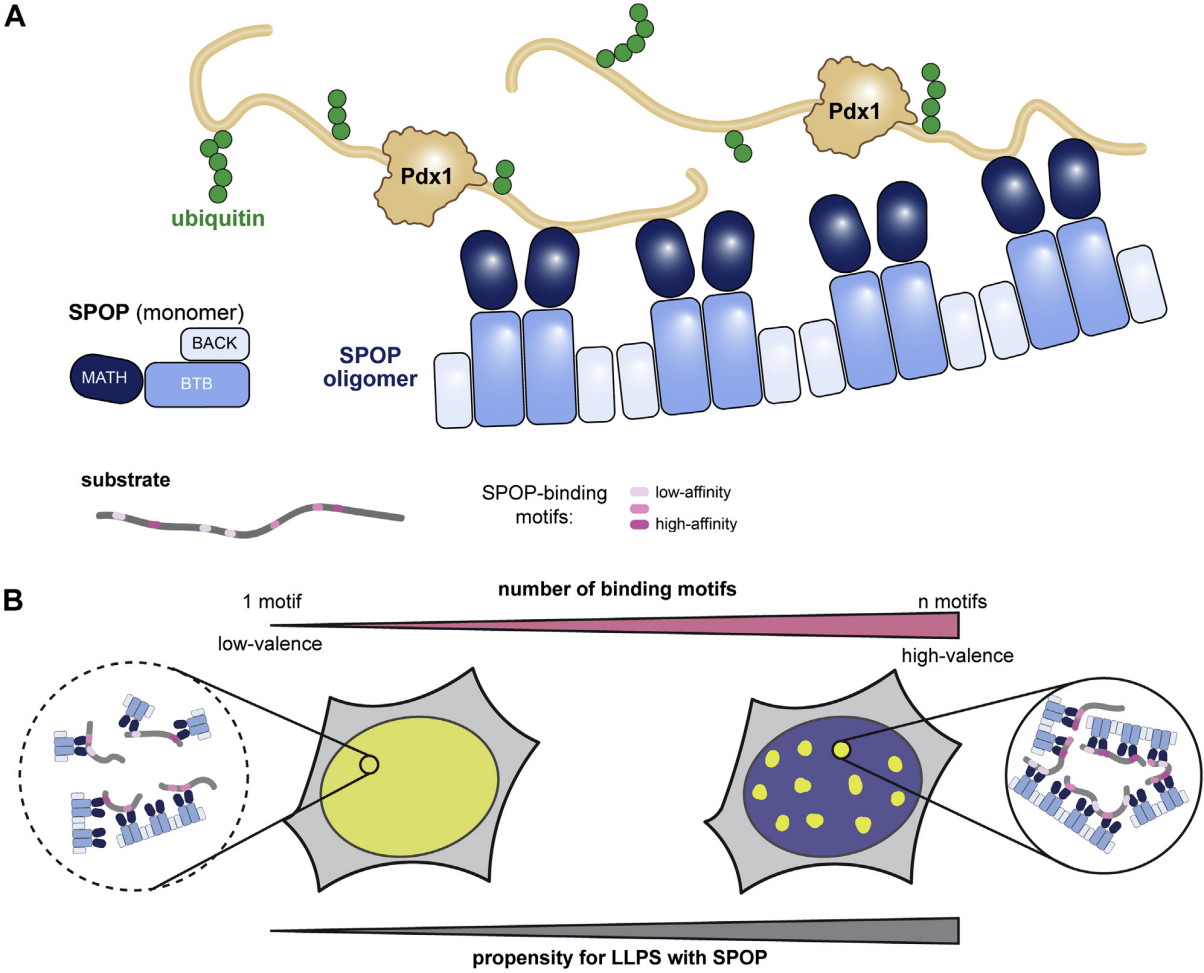
**Investigation of SPOP and Pdx1 behavior *in vitro* and in cells leads to new mechanistic insights into SPOP interactions.***A*, cartoon representation of SPOP and Pdx1 multivalent interaction and resultant ubiquitination. *B*, substrates may be categorized into high-valence (>3 SB motifs) or low-valence (<3 SB motifs) substrates. We propose that high-valence substrates are more prone to LLPS with SPOP *in vitro* and in cells, whereas low-valence substrates, such as Pdx1, rely on interaction with SPOP in the diffuse phase. Furthermore, the mode of number of substrates and mode of interaction between SPOP and substrate will, in part, dictate the degree of ubiquitination of the substrate. LLPS, liquid–liquid phase separation; Pdx1, pancreatic and duodenal homeobox 1; SB, SPOP-binding; SPOP, speckle-type POZ protein.

We propose that the valence of SPOP substrates determines their ability to phase separate with SPOP and that this binding mechanism determines the interaction fate in different subnuclear locations. A similar phenomenon, wherein LLPS propensity is dictated by the number of binding sites displayed by (*i.e.*, valence of) each binding partner, was demonstrated for Src homology 3 (Src, proto-oncogene tyrosine- protein kinase Src) domains binding to proline-rich motifs (30). As the number of tandem Src homology 3 domains and proline-rich motifs increased, so did the tendency of the system to phase separate; the phenomenon of valence as a contributor to phase separation has also been described computationally (42). High-valence substrates, such as DAXX or AR, permit phase separation mediated by multivalent interactions with SPOP and by substrate–substrate interactions (43). Low-valence substrates, such as Pdx1 or those with ∼three or fewer binding sites, may not support phase separation, but still permit high-affinity interaction with SPOP (Fig. 7*B*). In this way, some substrates may utilize a concentration thresholding regulatory mechanism whereby high levels of substrate are capable of relocalizing SPOP for their degradation.

In concert with previous studies, our findings allow us to propose that the interaction of SPOP and its numerous substrates is highly adaptable. First, we anticipate that other SPOP substrates or motifs within known substrates may exist that remain undetected because of deviations of their SB motif sequences from the established consensus motif. Furthermore, the amino acids that lie just outside the consensus motif in several substrates may play integral roles in facilitating a highly stable and specific SPOP–substrate interaction. Our finding that phase separation of SPOP with its substrate is not required for a functional interaction with Pdx1 in cells reveals another way in which SPOP may tailor its localization dependent on the substrate. Finally, our work supports the hypothesis that phase-separation behavior is likely linked to the number and affinity of binding sites presented by the substrate.

## Experimental procedures

### Cell lines

HeLa cells were cultured under sterile conditions with Dulbecco’s modified Eagle’s medium (DMEM) (Gibco) supplemented with 10% fetal bovine serum and antibiotics/antimycotics (Gibco) (see Table S5 for key reagents and resources). T-REx-293 cells (cat. no. R710-07) were obtained from Invitrogen. Cells were grown on DMEM supplemented with 10% fetal bovine serum (Gibco), GlutaMax (Gibco), and Penicillin-Streptomycin (Gibco) and kept under selection by 5 μg/ml blasticidin. All cells were grown at 37 °C under 5% CO_2_ and were mycoplasma free at the time the experiments were performed.

### Plasmids

An *Escherichia coli* codon-optimized DNA sequence encoding amino acids 205 to 283 of the human Pdx1 sequence (Pdx1-C) in the multiple cloning site of pET49b(+) was purchased from GeneArt (Thermo Fisher). The pET49b(+) backbone encodes for N-terminal glutathione S-transferase and 6X His tags, followed by a 3C protease recognition motif. In all experiments described herein, a mutant containing an exogenous tryptophan at the N terminus of the human Pdx1-C sequence was used for the purposes of quantitation. Mutations in Pdx1-C were generated by Q5 site-directed mutagenesis (New England Biolabs) with primers designed on NEBbasechanger.com according to the manufacturer protocol. Similarly, an *E. coli*–optimized DNA sequence encoding residues 28 to 166 of human SPOP (SPOP–MATH) was purchased in pET49b(+) from GeneArt. Notably, the constructs within pET vectors contain N-terminal Gly–Pro–Gly artifacts after treatment with 3C protease. A plasmid encoding His–SUMO–SPOP human residues 28 to 359 harboring a tobacco etch virus protease recognition site directly N terminal to the beginning of the native SPOP protein sequence was generated previously (21).

A plasmid encoding full-length human Pdx1 (amino acids 1-283) in pcDNA3.1 with a C-terminal GFP tag was purchased from GenScript. Full-length mouse SPOP (identical to the human sequence) with a V5 epitope tag in pcDNA3 was generated previously (21). The coding sequence for full-length mouse SPOP was inserted into pcDNA4/TO/myc (Invitrogen) for the T-REx system. pcDNA3-myc-CUL3 (cat no. 19893) and pcDNA–HA2–ROC1 (Rbx1) (cat no. 19897) were obtained from Addgene. pcDNA3 containing His-ubiquitin was a gift from Wenyi Wei.

### Protein expression and purification

BL-21(DE3) *E. coli* cells containing the GST–His–Pdx1-C plasmid were grown to an optical density of 0.6 to 0.8 at 600 nm in LB, at which point, expression was induced by the addition of 0.5 mM IPTG. After incubation at 37 °C for 3 h, cells were pelleted by centrifugation at 3700*g* and stored at −80 °C or lysed immediately. GST–His–SPOP–MATH was generated similarly, except expression was carried out at 15 °C for 18 h. Rosetta BL-21(DE3) cells containing His–SUMO–SPOP were grown in 5052-ZYM autoinduction media for 8 h at 37 °C followed by 15 °C for 18 h. Protein constructs for NMR were expressed in M9 minimal media supplemented with appropriate antibiotics, 1X minimal essential medium vitamins (Gibco), 1 mM MgSO_4_, 1X trace metals (Teknova), and NH_4_Cl and D-glucose. Uniform ^15^N enrichment was achieved by the use of ammonium chloride (^15^N, 99%, cat no. NLM–467–PK, Cambridge Isotope Laboratories, Inc) as the sole nitrogen source. Uniform ^15^N and ^13^C enrichment was achieved by the incorporation of ^15^N-ammonium chloride and D-glucose (U-^13^C_6_, 99%, cat no. CLM–1396–PK, Cambridge Isotope Laboratories, Inc). Isotopically enriched proteins were grown and expressed as described above.

Cell pellets of GST–His–SPOP–MATH or GST–His–Pdx1-C were resuspended in the lysis buffer (50 mM Tris HCl, pH 7.5, 500 mM NaCl, 20 mM imidazole, 5 mM β-mercaptoethanol) supplemented with 1X EDTA-free protease inhibitor cocktail (Millipore #539137) and 1 mM PMSF. Cells were lysed by sonication, and the lysate was clarified by centrifugation at 14,000*g* at 4 °C for 30 min. The clarified lysate was passed through a 5 μm syringe filter and applied to Ni-NTA resin (G-Biosciences) equilibrated with the lysis buffer. Bound proteins were washed with the lysis buffer supplemented with 0.1% Triton X-100 followed by the lysis buffer. Proteins were eluted from the column with the elution buffer (50 mM Tris HCl, pH 7.5, 500 mM NaCl, 200 mM imidazole, 5 mM β-mercaptoethanol). The Ni-NTA eluate was subjected to proteolytic cleavage by 1-mg 3C Protease per liter of culture under dialysis against 4 l of the lysis buffer for 16 h at 4 °C. Dialysates were applied to a second Ni-NTA column, and flow-through fractions were concentrated in a 3K MWCO centrifugal filter (Millipore).

The concentrated SPOP–MATH flow-through fraction was further purified by FPLC on a Sephacryl S100 gel filtration column in 50 mM Tris, pH 7.5, 150 mM NaCl, and 5 mM DTT at 4 °C. Fractions were analyzed by SDS-PAGE, and fractions containing SPOP–MATH were pooled, concentrated, and buffer-exchanged as necessary. The concentrated Pdx1-C flow-through fraction was heated at 70 °C for 10 min followed by centrifugation to pellet insoluble material. The supernatant was passed through a 0.2 μm centrifugal filter and then exchanged into ion-exchange buffer A (20 mM sodium phosphate, pH 5.3, 10 mM NaCl, 6 M urea). Pdx1-C was concentrated to <1 ml and applied to a 1-ml HiTrap Q HP anion-exchange column equilibrated in ion-exchange buffer A. The ion-exchange flow-through fraction was collected, concentrated, and buffer-exchanged as necessary. Contaminant proteins were eluted in ion-exchange buffer B (20 mM sodium phosphate, pH 5.3, 500 mM NaCl, 6 M urea).

Cells containing His–SUMO–SPOP were lysed by microfluidizer in the lysis buffer supplemented with 1X protease inhibitor cocktail and 1 mM PMSF. The lysate was clarified by centrifugation at 14,000*g* and then the supernatant was filtered and purified on a Ni-NTA column as described above. The Ni-NTA elution containing His–SUMO–SPOP was subjected to proteolytic cleavage by 1-mg tobacco etch virus protease per liter of expression culture under dialysis against 4 l of the lysis buffer for 60 h at 4 °C. The SPOP dialysate was passed over a second Ni-NTA column. The flow-through fraction from the second Ni-NTA column was concentrated and loaded (multiple injections) onto an Superdex 200 gel filtration column in 20 mM Tris, pH 7.5, 150 mM NaCl, and 5 mM DTT at 4 °C. Fractions were analyzed by SDS-PAGE, and those containing pure SPOP were pooled, concentrated, and buffer-exchanged as needed.

### Peptide synthesis and preparation

Solid-phase–synthesized peptides (Puc: ENLACDEVTSTTSSST, Pdx1–SBM1: Ac-GVAEPEQDCAVTSGEELLALPP, Pdx1–SBM1f: EQDCAVTSGEE, Pdx1–SBM2: Ac-LSASPQPSSVAPRRPQEPR, mock: Ac-GSSSEADEMAKALEAELNDLM) purified by HPLC were purchased from the Tufts University Core Facility. For NMR studies and FA, lyophilized peptides were resuspended in 50 mM sodium phosphate, pH 6.5, and 50 mM sodium chloride and quantified by FTIR spectroscopy. f-Puc ([FITC]-ENLACEDEVTSTTSSST) was purchased from GenScript and contains an N-terminal FITC tag for FA experiments. f-Puc was resuspended to a final concentration of 0.9 mM and used in PBS, pH 7.4.

### NMR data collection and analysis

NMR data were collected on Bruker Avance III 500, 600, or 850 MHz spectrometers equipped with TCI triple-resonance cryoprobes. All data collection and initial processing were carried out in TopSpin (Bruker). NMR experiments on all proteins were collected in 50 mM sodium phosphate, pH 6.5, 50 mM sodium chloride, 5 mM DTT, 0.01% sodium azide, and 5% D_2_O unless otherwise noted.

Backbone assignments of Pdx1-C were described previously (44) and have been deposited in the biological magnetic resonance bank (BMRB 19596). Resonance assignments of SPOP–MATH (residues 28–166 containing an N-terminal Gly–Pro–Gly cloning artifact) were generated by standard methods based on a sensitivity-enhanced ^1^H, ^15^N HSQC experiment (eight scans, 2048 × 256 complex data points) collected on approximately 0.6 mM ^13^C, ^15^N–SPOP–MATH. Three-dimensional experiments for assignments were collected according to the following: HNCO (eight scans, 2048 (^1^H) × 64 (^15^N) × 256 (^13^C), with sweep widths of 12 ppm, 32 ppm, and 22 ppm, respectively); HN(CA)CO (16 scans, 2048 (^1^H) × 64 (^15^N) × 256 (^13^C), with sweep widths of 12 ppm, 32 ppm, and 22 ppm, respectively); CBCA(CO)NH (eight scans, 2048 (^1^H) × 64 (^15^N) × 180 (^13^C), with sweep widths of 12 ppm, 32 ppm, and 75 ppm, respectively); HNCACB (16 scans, 2048 (^1^H) × 64 (^15^N) × 180 (^13^C), with sweep widths of 12 ppm, 32 ppm, and 75 ppm, respectively); and CC(CO)NH (16 scans, 2048 (^1^H) × 64 (^15^N) × 128 (^13^C), with sweep widths of 12 ppm, 32 ppm, and 75 ppm, respectively). Peak picking and assignment were carried out manually in SPARKY 3 or NMRFAM-Sparky (45, 46).

Addition of Pdx1 peptides into ^15^N–SPOP–MATH was monitored by sensitivity-enhanced ^1^H, ^15^N HSQC. The peptides were mixed in a 4:1 peptide:SPOP mole ratio with between 0.1 and 0.2 mM ^15^N–SPOP–MATH. Each experiment consisted of 16 scans and 2048 × 256 complex data points. Spectral comparison was carried out in NMRFAM-Sparky using the assigned ^15^N-SPOP-MATH spectrum as a reference. Integrated peak intensities before and after the addition of peptide were compared and normalized within each dataset, and the percent change (1 − I_bound_/I_unbound_) was plotted on the surface of published SPOP–MATH cocrystal structures (PDB IDs: 3IVV or 6F8F) (25, 33) using PyMol (PyMOL Molecular Graphics System, Version 2.1.1, Schrödinger, LLC). In each case, surface coloring corresponds to thresholds of intensity change, where the greatest resonance intensity changes are reflected by the lightest color. For Pdx1–SBM1 and Pdx1–SBM1/SBM2 experiments, the thresholds were set at percent change = >90 (lightest), 80 to 90, 70 to 80, and <70 (darkest). Residues that were overlapped, unassigned, or otherwise omitted from the analysis are binned and colored with the resonances exhibiting a percent change less than 0.7. The Pdx1–SBM2 titration was binned according to the following percent changes: >70 (lightest), 40 to 70, and <40 (darkest).

Addition of SPOP–MATH into ^13^C, ^15^N–Pdx1-C was monitored by ^13^C direct-detect (HACA)CON experiments (44) consisting of 16 scans and 1024 × 128 complex data points. SPOP–MATH was added to ^13^C, ^15^N–Pdx1-C such that the final concentrations were 0.35 mM and 0.37 mM, respectively. Peaks from each spectrum were integrated and used to calculate a percent intensity change in Pdx1-C resonances after binding of SPOP–MATH. SPOP_28–359_ was added to ^13^C, ^15^N–Pdx1-C at a 4-fold molar excess and detected by HSQC or (HACA)CON. Line broadening precluded residue-specific analysis of SPOP_28–359_ and Pdx1-C.

### Fluorescence anisotropy

All FA experiments were conducted in 50 mM sodium phosphate, pH 6.5, 50 mM sodium chloride, 5 mM DTT, 1% BSA, and 0.01% Triton X-100. In all experiments, the source of fluorescence signal was f-Puc purchased from GenScript, at a final concentration of 40 nM. For direct FA measurements, SPOP–MATH or SPOP_28–359_ of concentrations between 0.001 and 300 μM was added to wells of 96- or 384-well plates containing f-Puc and buffer. For competition-mode experiments, serial dilutions of each competitor (Pdx1 peptide or recombinant protein) were prepared ranging from ∼0.01 μM to ∼2 mM (concentration range varied based on peptide solubility) in wells of 96- or 384-well plates containing 40 nM f-Puc and 6 μM SPOP–MATH or SPOP_28–359_. The fluorescence polarization of each well was measured (from which FA was calculated) using either a TECAN Infinite M1000 Pro or BioTek Synergy 4 fluorometer. All experiments were performed with at least three technical replicates. FA data points were fit to a complete competitive binding model (23) to determine dissociation constants using in-house Python scripts (see Quantification and statistical analysis).

### Crystallography

Purified SPOP–MATH in 50 mM Tris, pH 7.5, and 50 mM NaCl was concentrated to 40 mg/ml (∼2.4 mM) and then mixed with Pdx1 SBM2 peptide (LSASPQPSSVAPRRPQEPR, human Pdx1 residues 265–283) to achieve a final mole ratio of 2:1 peptide:SPOP–MATH. The SPOP–peptide mixture was combined 1:1 by volume with a precipitant solution containing 0.08 M magnesium acetate, 0.05 M sodium cacodylate, pH 6.5, and 30% PEG-4000. The complex of SPOP–MATH bound with the Pdx1 SBM2 peptide crystallized within 1 week at room temperature (RT) (21 °C) by hanging-drop vapor diffusion. Crystals were harvested in the absence of a cryoprotectant solution. Separately, purified SPOP–MATH in 50 mM Tris, pH 7.5, and 50 mM NaCl was concentrated to 20 mg/ml (∼1.2 mM) and mixed with a precipitant solution containing 0.1 M Hepes, pH 7.5, 5 mM DTT, and 23% PEG-550 methyl ether. Unbound SPOP–MATH crystallized within 4 weeks at RT (21 °C) by hanging-drop vapor diffusion. Crystals were harvested in the presence of 20% glycerol as a cryoprotectant.

The SPOP–MATH–SBM2 (PDB ID: 7KPK) dataset was collected at 100 K at the Advanced Photon Source at Argonne National Laboratory at beamline 23-ID-B of the General Medical Sciences/Cancer Collaborative Access Team. The unbound SPOP–MATH (PDB ID: 7KPI) dataset was collected at 100 K at the Advanced Light Source at Lawrence Berkeley National Laboratory at beamline 8.2.2 funded by the Howard Hughes Medical Institute. Raw diffraction images were processed and scaled in HKL-2000 (47). Initial phases were obtained with molecular replacement with by using the PHASER-MR package in the CCP4 software suite (48, 49). PDB entry 3IVB (SPOP^MATH^–core histone Macro-H2ASBCpep1 complex) without coordinates for the peptide was used as the molecular replacement model (25). Models were manually adjusted in Coot (50) and refined iteratively in Refmac5 (51) within CCP4. Data collection and refinement statistics are provided for both structures in Table S2. All-atom contacts and geometry for both structures were analyzed by MolProbity (52). Structure figure generation, RMSD calculations, and distance measurements were carried out in PyMOL (Schrödinger, LLC). Hydrogen bond contacts and their distances are given in Table S3.

Crystals of both unbound SPOP–MATH and SPOP–MATH–SBM2 belong to the C121 space group, and each has a single monomer in the asymmetric unit. The final model of unbound SPOP–MATH consists of human SPOP residues 28 to 119 and 121 to 165 with an exogenous “PG” N-terminal to the native sequence belonging to a cloning artifact. The model of unbound SPOP–MATH also contains 146 water molecules. The final model of SPOP–MATH–SBM2 consists of human SPOP residues 28 to 43, 49 to 119, and 121 to 165 with an exogenous “PG” N-terminal to the native sequence belonging to a cloning artifact in chain A. Human Pdx1 residues 267 to 273 are modeled in chain B, and 108 water molecules are modeled in chain S. Of the residues modeled in both structures, 100% are in the allowed or generously allowed regions of the Ramachandran plot (Table S2).

### Immunofluorescence

For immunofluorescence experiments, 0.5 × 10^6^ cells were seeded on borosilicate glass coverslips in 6-well plates. After 24 h, cells were transfected with 400 ng of each plasmid using the Qiagen Effectene transfection reagent according to the manufacturer protocol. 24 h after transfection, the cells were treated with 4% paraformaldehyde in PBS for 30 min at RT. Cells were permeabilized with 0.1% Triton X-100 in PBS (PBST) for 5 min at 21 °C, followed by blocking in 0.5% BSA in PBST for 1 h at 21 °C. Signal from GFP-tagged proteins was observed directly. Cells were stained in primary antibodies diluted in 0.5% BSA in PBST overnight at 4 °C with mouse anti-SC-35 (1:300, ab11826; Abcam) and chicken anti-V5 (1:300, NB600-379; Novus Biologicals). Cells were incubated with secondary antibodies for 2 h at 21 °C. The secondary antibodies used were goat anti-mouse Alexa Fluor 647 (1:250, A-21235; Thermo Fisher) and goat anti-chicken Alexa Fluor 555 (1:250, A-21437; Thermo Fisher). Cells on coverslips were treated with 300 nM 4′,6-diamidino-2-phenylindole (DAPI) for 5 min at 21 °C. Coverslips were mounted on glass slides with ProLong Diamond mounting reagent (Thermo Fisher) and cured before imaging on a Zeiss LSM 880 Upright microscope. All images were analyzed and prepared in Fiji (53).

### Ubiquitination and Western blots

T-REx-293 cells (Invitrogen) were seeded at a density of 0.8 × 10^6^ per well in 6-well plates (tissue culture–treated plates (cat. no 3516, Corning)). After 24 h, cells were transfected with plasmids encoding Pdx1–GFP, SPOP–myc, His–ubiquitin, HA–Rbx1, and myc–CUL3 (as indicated) using the Qiagen Effectene transfection reagent. 24 h after transfection, the cells were treated with either dimethyl sulfoxide or 20 μM MG132 for 4 h before being harvested. Cells were lysed in buffer A (6 M guanidine HCl, 100 mM sodium phosphate, pH 8.0, and 10 mM imidazole). The lysates were sonicated, cleared, and incubated with Ni-NTA Agarose (Qiagen) for 3 h at RT, followed by 16 h at 4 °C. The beads were washed twice with buffer A and twice with a buffer composed of 1:3 buffer A:buffer T1 (buffer T1: 25 mM Tris HCl, pH 8.0, and 20 mM imidazole). The beads were finally transferred to buffer T1 and then boiled in the SDS-PAGE loading buffer containing 300 mM imidazole. Pdx1 stability assays were performed as described above, except that the cells were not treated with MG132. Cells expressing Pdx1–GFP or a variant were harvested 28 h after transfection, resuspended in 2x SDS-loading dye, and boiled for 5 min before SDS-PAGE analysis.

All proteins were resolved by SDS-PAGE. Separated proteins were analyzed by immunoblotting using either the ECL or the ECL Select Western blotting detection reagents (Amersham). The antibodies used were mouse anti-Pdx1 used at 1:500 for pulldown or 1:2000 for inputs (clone 26771, MAB2419, R&D Systems), mouse anti-myc (1:250, clone 9E10, Santa Cruz Biotechnology), and rabbit anti-GAPDH (1:8000, clone EPR1689, Abcam). horseradish peroxidase-conjugated secondary antibodies were sourced from Jackson ImmunoResearch Laboratories and used at 1:20,000 dilution (goat anti-rabbit IgG, 111-035-003 and goat anti-mouse, 115-035-003).

### Quantification and statistical analysis

#### *K*_D_ determination from fluorescence anisotropy

Representative data from FA titrations are shown in Figure 4. All titrations were performed in triplicate for rigor. Direct FA titrations were performed to determine the dissociation constants of the fluorescent probe toward SPOP constructs. Direct titration data were fit according to equation 6 in (54). Competition mode titration data were fit according to the fitting function in (23). Average dissociation constants and standard error from the mean for all constructs from competition experiments may be found in Table S4. The python scripts used in plotting and fitting may be found at https://github.com/idpemery/spop-pdx1.

#### Analysis of immunofluorescence data

All immunofluorescence data were averaged over three biological replicates. Cells not expressing both Pdx1 and SPOP were excluded from sampling. Raw images were collected in the Zeiss Zen software and then exported for viewing and analysis in Fiji. Nuclei of individual cells were designated manually in Fiji using the DAPI channel as a guide. The cells were binned based on whether SPOP was localized to nuclear speckles (puncta); cells with both diffuse and punctate SPOP signal were binned as “speckled.” The grayscale signal intensity of the green channel (Pdx1–GFP) was averaged over the DAPI-designated nuclear area. Next, the raw GFP intensity data points (one data point per cell analyzed) were plotted to generate a violin plot distribution. The average, minimum, and maximum values are shown by horizontal lines on the plot. Individual data points are also shown for rigor. A two-tailed Student’s *t* test was performed on Pdx1 variants that resulted in two distinct SPOP localization patterns (diffuse *versus* speckled) to determine statistical significance. All plotting and statistical tests were performed using in-house python scripts that can be found at https://github.com/idpemery/spop-pdx1.

## Data availability

The crystal structures reported in this publication have been deposited in and validated by the PDB. The structure of unbound SPOP–MATH is assigned PDB ID: 7KPI. The structure of SPOP–MATH in complex with a Pdx1 fragment is assigned PDB ID: 7KPK. Python scripts used in data analysis are provided with example files at https://github.com/idpemery/spop-pdx1.

## Supporting information

This article contains supporting information (21, 22, 23, 36, 46, 47, 48, 49, 50, 51, 52, 53, 56, 57, 58, 59).

## Conflicts of interest

The authors declare that they have no conflicts of interest with the contents of this article.
