# Supplementary material for: Intrinsically disordered substrates dictate SPOP subnuclear localization and ubiquitination activity
Source: J Biol Chem. 2021 Apr 22;296:100693. doi: 10.1016/j.jbc.2021.100693 (PMC8138767; doi:10.1016/j.jbc.2021.100693)
Supplement: Supplemental Figures S1–S3 and Tables S1–S5 [file mmc1.pdf]

Supporting Information

Intrinsically disordered substrates dictate SPOP subnuclear localization and ubiquitination activity

Emery T. Usher, Nafiseh Sabri, Roman Rohac, Amie K. Boal, Tanja Mittag, Scott A. Showalter

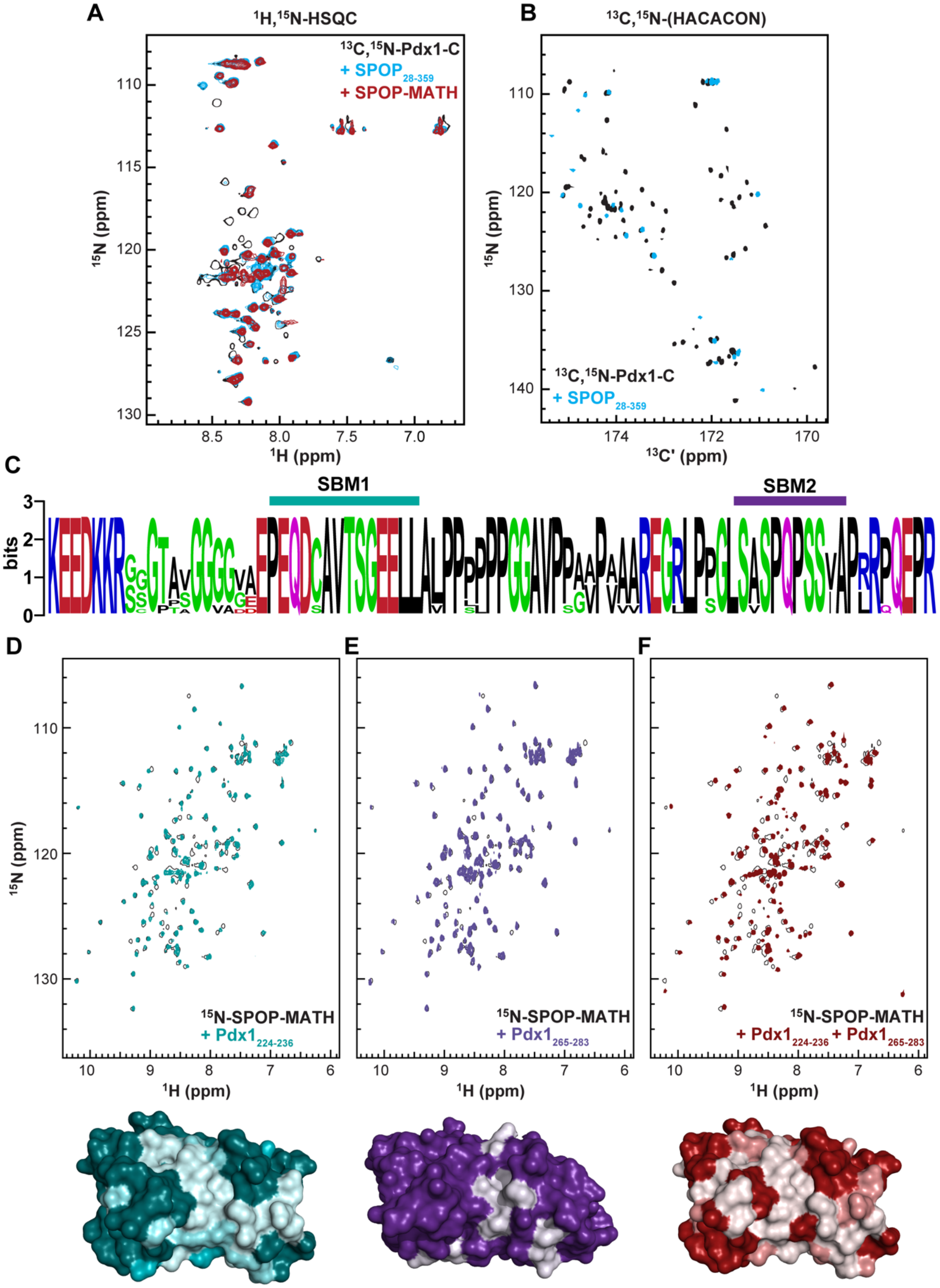
A
$^1H,^{15}N$-HSQC
$^{13}C,^{15}N$-Pdx1-C
+ $SPOP_{28-359}$
+ SPOP-MATH
^{15}N (ppm)
^1H (ppm)
B
$^{13}C,^{15}N$-(HACACON)
$^{13}C,^{15}N$-Pdx1-C
+ $SPOP_{28-359}$
^{15}N (ppm)
$^{13}C'$ (ppm)
C
SBM1
SBM2
bits
D
E
F
^{15}N-SPOP-MATH
+ $Pdx1_{224-236}$
^{15}N-SPOP-MATH
+ $Pdx1_{265-283}$
^{15}N-SPOP-MATH
+ $Pdx1_{224-236}$ + $Pdx1_{265-283}$
^{15}N (ppm)
^1H (ppm)

Figure S1: Pdx1-C contains two motifs that interact with SPOP-MATH (related to Figure 3).

(A) ^{1}H, ^{15}N HSQC spectrum of Pdx1-C (hollow black resonances) with the addition of either one molar equivalent of SPOP-MATH (red resonances) or four molar equivalents of $SPOP_{28\text{-}359}$ (cyan resonances). With the exception of a few peaks, the spectra of SPOP-MATH-bound Pdx1-C and $SPOP_{28\text{-}359}$-bound Pdx1-C are highly comparable.
(B) ^{13}C, ^{15}N (HACA)CON spectrum of Pdx1-C (hollow black resonances) with the addition of 4 molar equivalents of $SPOP_{28\text{-}359}$ (cyan resonances). Intensity change analysis using $SPOP_{28\text{-}359}$-bound Pdx1-C was not pursued due to substantial line broadening when oligomeric SPOP was introduced to Pdx1-C.
(C) Weblogo representing the multiple sequence alignment of Pdx1 C-terminal IDRs from *H. sapiens, M. musculus, M. auratus, R. norvegicus, G. gorilla, A. melanoleuca, P. troglodytes, and P. paniscus.* The SB motif sequences are denoted above by teal and purple bars. The weblogo graphic was generated using the T-Coffee multiple sequence alignment tool (56).
(D) Overlaid NMR spectra of SPOP-MATH alone (hollow black resonances) and four molar equivalents of a peptide corresponding to Pdx1-SBM1 (teal resonances). The per residue intensity change is mapped on the surface of SPOP-MATH (absent Puc peptide) from PDB ID: 3IVV.
(E) Overlaid NMR spectra of SPOP-MATH alone (hollow black resonances) and a peptide corresponding to four molar equivalents of Pdx1-SBM2 (purple resonances). SPOP-MATH resonance intensity changes are plotted as described in (D). SBM2 addition leads to fewer intensity changes, but those that are observed map well to the canonical substrate-binding groove.
(F) Overlaid NMR spectra of SPOP-MATH alone (hollow black resonances) and four molar equivalents of both SBM1 and SBM2 peptides. SPOP-MATH resonance intensity changes are plotted as described in (D) and largely resemble the binding of SBM1 alone, suggesting that a direct competition between the two Pdx1 SB motifs allows preferential binding of the stronger peptide (SBM1).

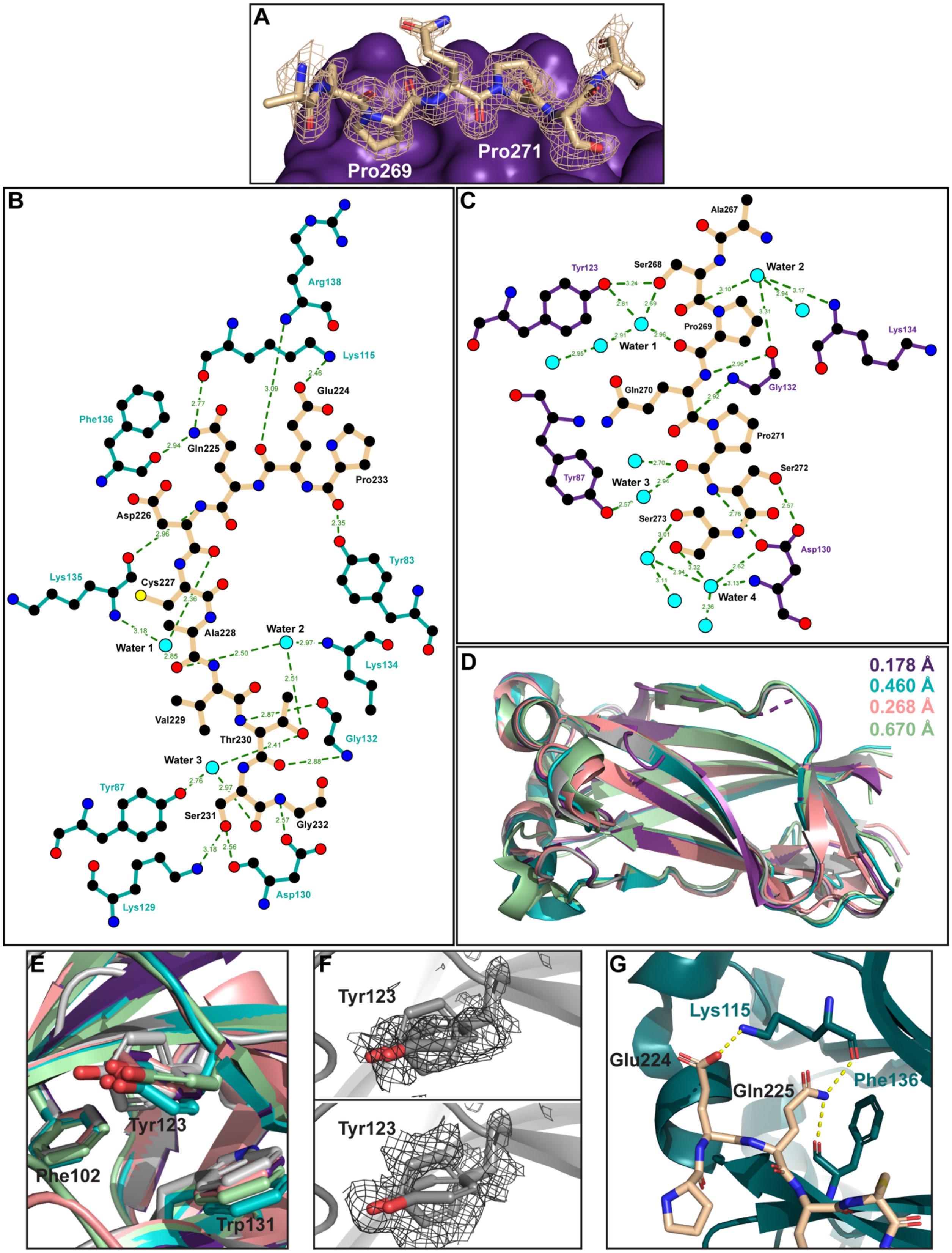
A
Pro269
Pro271
B
Arg138
Lys115
Glu224
Phe136
Gln225
Pro233
Asp226
Tyr83
Lys135
Cys227
Ala228
Water 1
Water 2
Lys134
Val229
Thr230
Gly132
Water 3
Tyr87
Ser231
Gly232
Lys129
Asp130
C
Ala267
Tyr123
Ser268
Water 2
Pro269
Lys134
Water 1
Gly132
Gln270
Pro271
Ser272
Tyr87
Water 3
Ser273
Asp130
Water 4
D
0.178 Å
0.460 Å
0.268 Å
0.670 Å
E
Tyr123
Phe102
Trp131
F
Tyr123
Tyr123
G
Lys115
Glu224
Phe136
Gln225

Figure S2: Structural characterization of Pdx1-SPOP leads to new insights about broader SPOP function (related to Figure 4).

(A) Omit map of Pdx1-SBM2 peptide contoured at + 3σ (mesh). The electron density clearly conveys the location and orientation of the proline residues (P269 and P271) in the SBM2-SPOP-MATH structure.
(B) Hydrogen bonding network of Pdx1-SBM1 to SPOP-MATH (from PDB ID: 6F8F). Specific contacts are tabulated in Table S3.
(C) Hydrogen bonding network of Pdx1-SBM2 to SPOP-MATH (this paper). Diagrams were made in LigPlot+ (57). Specific contacts are tabulated in Table S3.
(D) Alignment of SPOP-MATH chains from several co-crystal structures. Peptides were deleted from the structures for display purposes. Each structure was aligned to the structure of unbound SPOP-MATH (gray) and the all-atom RMSD for each was calculated in PyMol. Structures are color-coded as follows: Pdx1-SBM2 (purple, this work), Pdx1-SBM1 (teal, 6F8F), Puc (pink, 3IVV), MacroH2A (green, 3IVB).
(E) Analysis of aligned SPOP-MATH chains bound to various substrates shows a 90-degree rotation of the side chain of Tyr123 between the unbound and bound structures (colored according to (D)).
(F) The difference map (contoured at +1.5σ) generated from unbound SPOP-MATH modeled with only one Tyr123 conformation shows positive density orthogonal to the conformation that is adopted in all bound-state structures. Two conformations of the Tyr123 side chain with a difference map are shown (top and bottom). This residue-specific change may permit peptide occupancy in the hydrophobic pocket and/or be coupled to the expulsion of water from pocket upon binding.
(G) Glu224 and Gln225 of Pdx1 lie upstream of the established SBM consensus sequence and make specific contacts with Lys115 on SPOP-MATH in the SBM-SPOP-MATH structure (PDB ID: 6F8F).

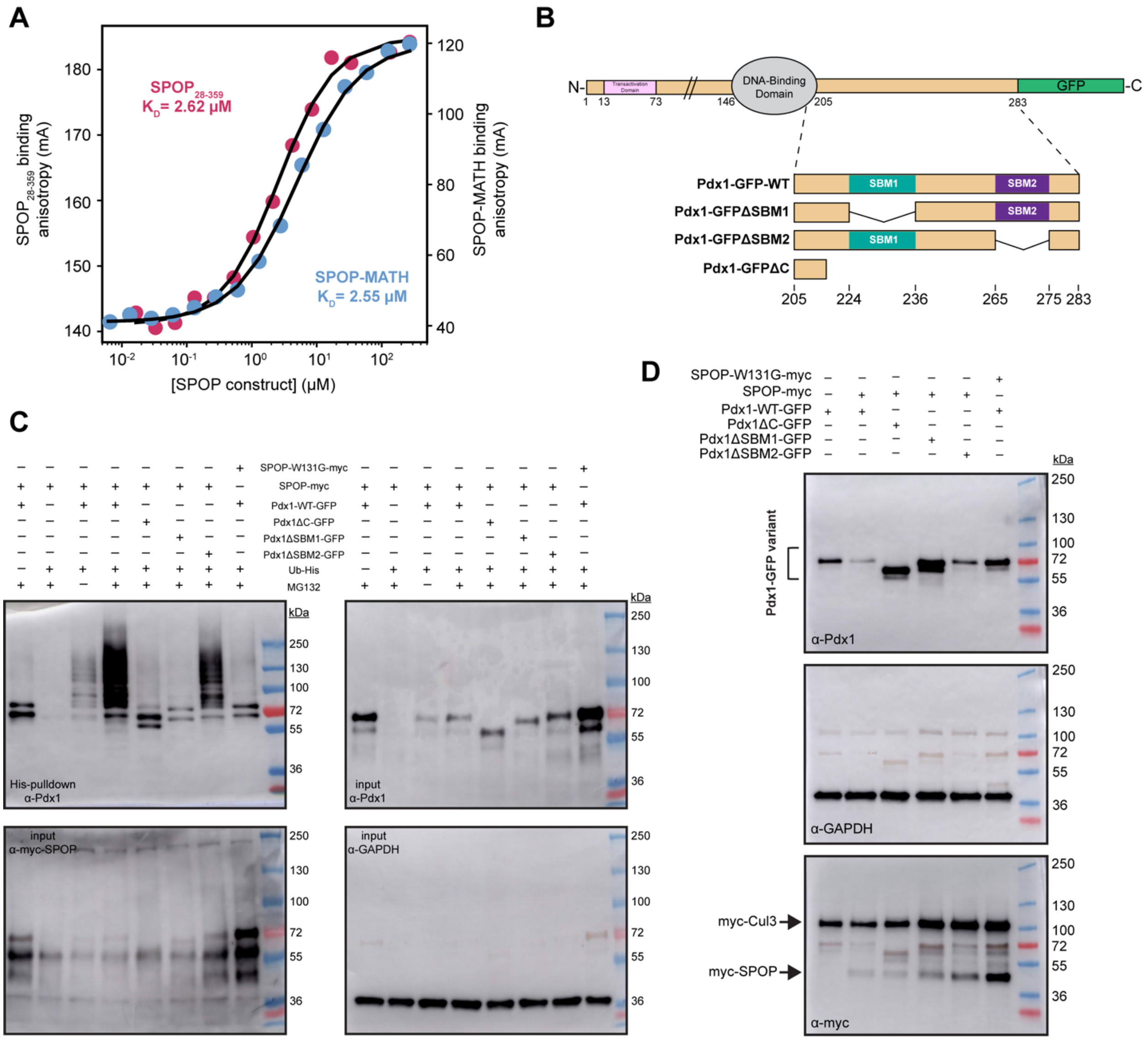

Figure S3: SPOP-Pdx1 interaction leads to polyubiquitination of Pdx1 (related to Figures 5 and 6).

(A) Direct titration of SPOP-MATH (blue) and SPOP$_{28-369}$ (pink) toward f-Puc. Dissociation constants were extracted from the fitted data (see Methods). SPOP-MATH bound to f-Puc with a dissociation constant of 2.62 µM. SPOP$_{28-369}$ binds to f-Puc with a dissociation constant of 2.55 µM.
(B) Domain architecture of GFP-Pdx1 variants used in this study. Importantly, no lysine residues were deleted in any variants to ensure that ubiquitination activity was not biased by the number of available lysines.
(C) Uncut multiplexed Western blots corresponding to Figure 6c.
(D) Pdx1 stability is dependent on the presence or absence of the SB motifs. T-REx cells were transfected with Pdx1-GFP variants and Pdx1 protein levels were assessed by Western blot after ~28 hours. Pdx1-GFP-WT is almost entirely degraded, whereas Pdx1-GFP lacking both SB motifs persists on the timescale of the experiment. Pdx1-

ΔSBM1 is markedly more stable than the WT and Pdx1-ΔSBM2 is still degraded to an extent but is slightly more stable than WT. Note that myc-SPOP migrates between 36 and 55 kDa and myc-Cul3 migrants just above 100 kDa.

Table S1: Pdx1 mutations found in patients with pancreas-linked diseases.

Mutation	Disease
C18R*	Type 2 Diabetes
P33T*	MODY4
Q59L*	Type 2 Diabetes
P63 frameshift (resulting in truncation)*	Pancreatic agenesis, MODY4
D76N*	Type 2 Diabetes, MODY3
E178G	Neonatal Diabetes
R197H	Type 2 Diabetes
G212R*	MODY3
E224K*	MODY4
P239Q*	MODY3
In-frame proline insertion after P242*	Type 2 Diabetes

*mutations found in the Pdx1 IDRs

Table S2: X-ray data collection and refinement statistics.

	SPOP-MATH	Pdx1SBM2/SPOP-MATH
**Data Collection**		
PDB ID	7KPI	7KPK
Wavelength (Å)	0.999995	0.799898
Space group	*C121*	*C121*
Cell dimensions		
a, b, c (Å)	62.64, 53.25, 41.60	61.80, 53.98, 41.56
α, β, γ (°)	90.00, 101.39, 90.00	90.00, 101.43, 90.00
Resolution (Å)	40.78-1.70 (1.73-1.70)‡	40.30-1.71 (1.73-1.71)
R_{merge}	0.046 (0.316)	0.045 (0.161)
I/σ	46.0 (8.7)	20.5 (7.7)
$CC_{1/2}$	(0.98)	(0.95)
Completeness (%)	96.9 (93.5)	87.6 (81.9)
Redundancy	7.6 (7.7)	3.4 (3.0)
**Refinement**		
No. reflections	14,542	12,920
R_{free} test set	730 (5.02%)	649 (5.02%)
R_{work}/R_{free}	0.160/0.196	0.181/0.227
No. atoms	1,335	1,238
Protein	1,189	1,084
Peptide	--	46
Water	146	108
Average B (Å^2)	18.0	18.0
Solvent B (Å^2)	49.9	52.4
R.M.S. deviations		
Bond length (Å)	0.011	0.014
Bond angle (°)	1.20	1.30

Ramachandran favored/allowed (%)	98.55/1.45	97.74/2.26
Clashscore, all (%ile)	5.04 (94%)	5.74 (92%)

‡values in parentheses refer to the high-resolution shell

Table S3: Select polar contacts between Pdx1-SBMs and SPOP-MATH.

	**Pdx1 Residue**	**SPOP Residue**	**Water molecule**	**Distance (Å)**
**SBM1**	P223 (bb)	Y83 (sc)		2.3
	E224 (sc)	K115 (sc)		2.5
	Q225 (sc)	K115 (bb)		2.8
	D226 (bb)	K125 (bb)		3.4
	D226 (bb)		Water 1	2.4
		K135 (bb)	Water 1	3.2
	A228 (bb)		Water 1	2.9
	A228 (bb)		Water 2	2.5
		K134 (bb)	Water 2	3.0
	T230 (sc)		Water 3	2.5
	S231 (bb)		Water 3	3.0
		Y87	Water 3	2.8
	S231 (sc)	K129		3.2
**SBM2**	S268 (sc)		Water 1	2.9
	S268 (sc)	Y123 (sc)	Water 1	3.2
		Y123 (sc)	Water 1	2.8
	S268 (bb)		Water 2	3.6
		K134 (bb)	Water 2	2.9
		G132 (bb)	Water 2	3.2
	Q270 (bb)	G132 (bb)		3.0
	P271 (bb)		Water 3	3.0
		Y87 (sc)	Water 3	2.6
	S272 (bb)	D130 (sc)		2.8
	S272 (sc)	D130 (sc)		2.6
		D130 (sc)	Water 4	2.6
	S273 (sc)		Water 4	3.4

Table S4: Binding affinities of Pdx1 toward SPOP-MATH and $SPOP_{28-359}$.

Competitor	Residue #s	Sequence	K_D (μM)[†]
		**Binding to SPOP-MATH**	
Puc	91-106	ENLACDEVTSTTSSST	2.6 ± 0.4
Pdx1-C	204-283	recombinant protein (see STAR)	32 ± 5
Pdx1-SBM1f	224-232	EQDCAVTSGEE	140 ± 55
Pdx1-SBM2	265-283	LSASPQPSSVAPRRPQEPR	630 ± 80
Mock (Fcp1)	941-961	GSSSEADEMAKALEAELNDLM	no binding
		**Binding to $SPOP_{28-359}$**	
Puc	91-106	ENLACDEVTSTTSSST	2.6 ± 1.0
Pdx1-C	204-283	recombinant protein (see STAR)	3.5 ± 1.5

[†]Dissociation constants reported in μM; error shown is the standard deviation from the mean of three independent replicates

Table S5: Key reagents and resources (see Experimental Procedures)

Reagent/Resource	Source	Identifier
Experimental Models: Cell Lines		
HeLa	Xin Zhang, Penn State	N/A
T-REx-293	Invitrogen	Cat# R710-07; RRID:CVCL_D585
Recombinant DNA		
pET49b-Pdx1C	(36)	N/A
pET49b-SPOP MATH (28-166)	This paper	N/A
pBAD-SPOP (28-359)	(21)	N/A
pET28a-3CPro	Linda Nicholson	N/A
pMal-C2-TEV S219V	(58)	Addgene #8827; RRID:Addgene_8827
pcDNA3.1-Pdx1-eGFP	This paper	N/A
pcDNA3.1-Pdx1ΔSBM1-eGFP	This paper	N/A
pcDNA3.1-Pdx1ΔSBM2-eGFP	This paper	N/A
pcDNA3.1-Pdx1ΔC-eGFP	This paper	N/A
pcDNA3-V5-SPOP	(21)	N/A
pcDNA3-V5-SPOP W131G	(23)	N/A
pcDNA-His6-Ubiquitin	Wenyi Wei	N/A
pcDNA3-HA2-ROC1 (Rbx1)	(59)	Addgene #19897; RRID:Addgene_19897
pcDNA3-myc-CUL3	(59)	Addgene #19893; RRID:Addgene_19893
pcDNA4/OT/myc-SPOP	This paper	N/A
Antibodies		

Chicken Polyclonal anti-V5	Novus Biologicals	Cat# NB600-379; RRID:AB_10003214
Mouse Monoclonal anti-SC-35	Abcam	Cat# ab11826; RRID:AB_298608
Goat Polyclonal anti-Chicken IgG, AF555-conjugated	Invitrogen	Cat# A21437; RRID:AB_1500593
Goat Polyclonal anti-Mouse IgG, AF647-conjugated	Invitrogen	Cat# A21235; RRID:AB_2535804
Mouse Monoclonal anti-Pdx1	R&D Systems	Cat# MAB2419; RRID:AB_10548285
Mouse Monoclonal anti-myc	Santa Cruz Biotechnology	Cat# 9E10
Rabbit Monoclonal anti-GAPDH	Abcam	Cat# EPR1689
Goat Polyclonal anti-Mouse IgG	Jackson ImmunoResearch Laboratories	Cat# 111-035-003
Goat Polyclonal anti-Rabbit IgG	Jackson ImmunoResearch Laboratories	Cat# 115-035-003
Oligonucleotides		
Pdx1-GFP aa224-236 deletion (ΔSBM1) FWD: GCACTTCCTCCGCCTCCA		
Pdx1-GFP aa224-236 deletion (ΔSBM1) REV: AGGCTCAGCAACTCCGCC		
Pdx1-GFP aa262-275 deletion (ΔSBM2) FWD: CCTAGACGGCCTCAAGAGCCCCGG		
Pdx1-GFP aa262-275 deletion (ΔSBM2) REV: GCCAGGAGGCAATCTGCCTTCTCTGGC		
Pdx1-GFP aa220-283 deletion (ΔC) FWD: GGATCCACTAGTCCAGTGTG		
Pdx1-GFP aa220-283 deletion (ΔC) REV: AACTCCGCCACCTCCAAC		
Chemicals, Enzymes, and Other Reagents		
ammonium chloride (^{15}N, 99%)	Cambridge Isotope Laboratories, Inc.	Cat# NLM-467-PK
D-glucose (U-$^{13}C_6$, 99%)	Cambridge Isotope Laboratories, Inc.	Cat# CLM-1396-PK
f-Puc: (FITC)-ENLACDEVTSTTSSSSST-NH2	Genscript	(22)
Software		

NEBaseChanger	https://nebasechanger.neb.com	
FIJI	(53)	http://fiji.sc; RRID:SCR_002285
Zen software	http://www.zeiss.com/corporate/en_de/global/home.html	RRID:SCR_013672
TopSpin	https://www.bruker.com/products/mr/nmr/nmr-software/nmr-software/topspin/overview.html	RRID:SCR_014227
NMRFAM_Sparky	(46)	https://nmrfam.wisc.edu/nmrfam-sparky-distribution/
Spyder	https://www.spyder-ide.org	RRID:SCR_017585
HKL-2000	(47)	https://www.hkl-xray.com; RRID:SCR_015547
CCP4	(48)	https://www.ccp4.ac.uk; RRID:SCR_007255
Phaser-MR	(49)	https://www.phenix-online.org/documentation/reference/phaser_mr.html; RRID:SCR_014219
Coot	(50)	https://www2.mrc-lmb.cam.ac.uk/personal/pemsley/coot/; RRID:SCR_014222
REFMAC5	(51)	RRID:SCR_014225
PyMOL	https://pymol.org/2/	RRID:SCR_000305
MolProbity	(52)	http://molprobity.biochem.duke.edu; RRID:SCR_014226
Ligplot+	(57)	https://www.ebi.ac.uk/thornton-srv/software/LigPlus/; RRID:SCR_018249
T-Coffee	(56)	http://tcoffee.crg.cat; RRID:SCR_011818
